# Transcriptional landscape of bone marrow-derived very small embryonic-like stem cells during hypoxia

**DOI:** 10.1186/1465-9921-12-63

**Published:** 2011-05-10

**Authors:** Sina A Gharib, Abdelnaby Khalyfa, Magdalena J Kucia, Ehab A Dayyat, Jinkwan Kim, Heather B Clair, David Gozal

**Affiliations:** 1 Center for Lung Biology and Division of Pulmonary and Critical Care Medicine, Department of Medicine, University of Washington, Seattle, WA, USA; 2 Department of Pediatrics, University of Louisville, Louisville, KY, USA; 3 Department of Pediatrics, University of Chicago, Chicago, IL, USA; 4 Stem Cell Biology Institute, University of Louisville, Louisville, KY, USA

## Abstract

**Background:**

Hypoxia is a ubiquitous feature of many lung diseases and elicits cell-specific responses. While the effects of hypoxia on stem cells have been examined under *in vitro *conditions, the consequences of *in vivo *oxygen deprivation have not been studied.

**Methods:**

We investigated the effects of *in vivo *hypoxia on a recently characterized population of pluripotent stem cells known as very small embryonic-like stem cells (VSELs) by whole-genome expression profiling and measuring peripheral blood stem cell chemokine levels.

**Results:**

We found that exposure to hypoxia in mice mobilized VSELs from the bone marrow to peripheral blood, and induced a distinct genome-wide transcriptional signature. Applying a computationally-intensive methodology, we identified a hypoxia-induced gene interaction network that was functionally enriched in a diverse array of programs including organ-specific development, stress response, and wound repair. Topographic analysis of the network highlighted a number of densely connected hubs that may represent key controllers of stem cell response during hypoxia and, therefore, serve as putative targets for altering the pathophysiologic consequences of hypoxic burden.

**Conclusions:**

A brief exposure to hypoxia recruits pluripotent stem cells to the peripheral circulation and actives diverse transcriptional programs that are orchestrated by a selective number of key genes.

## Background

Hypoxia is a pathophysiologic condition commonly seen in lung diseases and in many other disorders such as tissue ischemia and cancer [1]. In the developing embryo exposure to global reduction in tissue oxygen is a critical factor in recruitment and selective differentiation of embryonic stem cells that orchestrate organogenesis [2]. The presence of hypoxic microenvironments in adult tissues, as seen for example in solid tumors or during ischemic injury, can also lead to activation and recruitment of stem cells from specific tissue niches, including the bone marrow (BM) [3]. Under such local hypoxic circumstances, resident BM stem cells have been shown to become mobilized and facilitate the structural and functional repair of injured organs [4-6]. The effects of global hypoxia on stem cell activation and recruitment remain poorly understood despite being a common feature of many pulmonary disorders, and increasing evidence that BM-derived circulating progenitor cells may play a causative role in acute lung injury, pulmonary hypertension, pulmonary fibrosis and COPD [7-10].

Recently, Ratajczak *et al *isolated a homogenous population of rare, small stem cells from adult murine BM mononuclear cells that expressed embryonic lineage markers, and were thus named very small embryonic-like stem cells (VSELs) [11]. Pluripotency of these Sca-1^+ ^Lin^- ^CD45^- ^stem cells was established by demonstrating their ability to differentiate into cells representing all three germ layers [12]. Bone marrow-resident VSELs can be mobilized to the peripheral blood (PB) in response to specific chemoattractant gradients, including stromal derived factor-1 (SDF-1), hepatocyte growth factor (HGF), and leukemia inhibitory factor (LIF) [12,13]. Since SDF-1 is upregulated in response to hypoxia [14], it seemed plausible that chemokine-dependent recruitment of VSELs can occur during tissue injury characterized by hypoxia. Indeed, recent reports have demonstrated that these pluripotent stem cells are mobilized from BM to PB following organ damage associated with significant hypoxic burden, including ischemic stroke [15] and myocardial infarction [16].

Exposure to reduced oxygen tension causes widespread perturbations in cellular transcription [17] that is controlled by a number of critical regulators, most prominently hypoxia inducible factors (HIFs) [18]. While gene expression profiling of stem cells under various differentiating conditions has been undertaken [19], the transcriptional consequences of hypoxia in these cells is less studied. Recently, Westfall *et al *reported on the effects of 4% vs. 20% oxygen on gene expression of cultured human embryonic stem cells, and concluded that lower oxygen tension (4%) *in vitro *more accurately captures the true "physiologic" oxygen exposure *in vivo *by preserving the pluripotent property of these cells [20]. Indeed, a significant difficulty with all *in vitro *studies of hypoxic exposure is establishing the physiologically relevant oxygen tension during exposure [3,21] and retaining the *in vivo *undifferentiated state of stem cells [22].

To overcome these limitations, we utilized an *in vivo *murine model to systematically identify the transcriptional response of BM-derived VSELs to hypoxia. We hypothesized that exposing the animals to hypoxia will mobilize VSELs from their BM to peripheral circulation in a stem-cell specific chemokine gradient, and activate a spectrum of transcriptional programs that captures the diverse, pluripotent potential of these cells.

## Methods

### Animals

All animal experiments were performed according to protocols approved by the Institutional Animal Care and Use Committee (IACUC) of the University of Louisville. Eight-week-old, adult, male, C57BL/J6 mice were purchased from Jackson Laboratory (Bar Harbor, ME) and housed in a specific pathogen free environment.

### Hypoxic exposure

Mice were placed in identical commercially designed chambers (Oxycycler model A44XO; Biospherix, Redfield, NY, USA) that were operated under a 12:12-h light-dark cycle (6:00 a.m to 6:00 p.m.). Gas was circulated around each of the chambers, attached tubing, and other units at 10 l/min to maintain low ambient CO_2 _levels. An O_2 _analyzer measured the O_2 _concentration continuously and deviations from the desired concentration were corrected by addition of N_2 _or O_2 _through solenoid valves such that the moment-to-moment desired oxygen concentration of the chamber was programmed and adjusted automatically. Ambient CO_2 _in the chamber was monitored periodically and maintained at < 0.01% by adjusting overall chamber basal ventilation. Humidity was measured and maintained at 40-50% by circulating the gas through a freezer and silica gel. Ambient temperature was kept at 22-24°C. Hypoxic mice were exposed to F_i_O_2 _of 8%, a level that is associated with reproducible oxyhemoglobin saturations in the 75-80% range. Control animals were exposed to circulating room air (F_i_O_2_: 21%). Animals were exposed to hypoxia or normoxia for a period of 24 h and sacrificed immediately afterwards.

### Isolation of VSELs from bone marrow and peripheral blood

**Figure 1:**
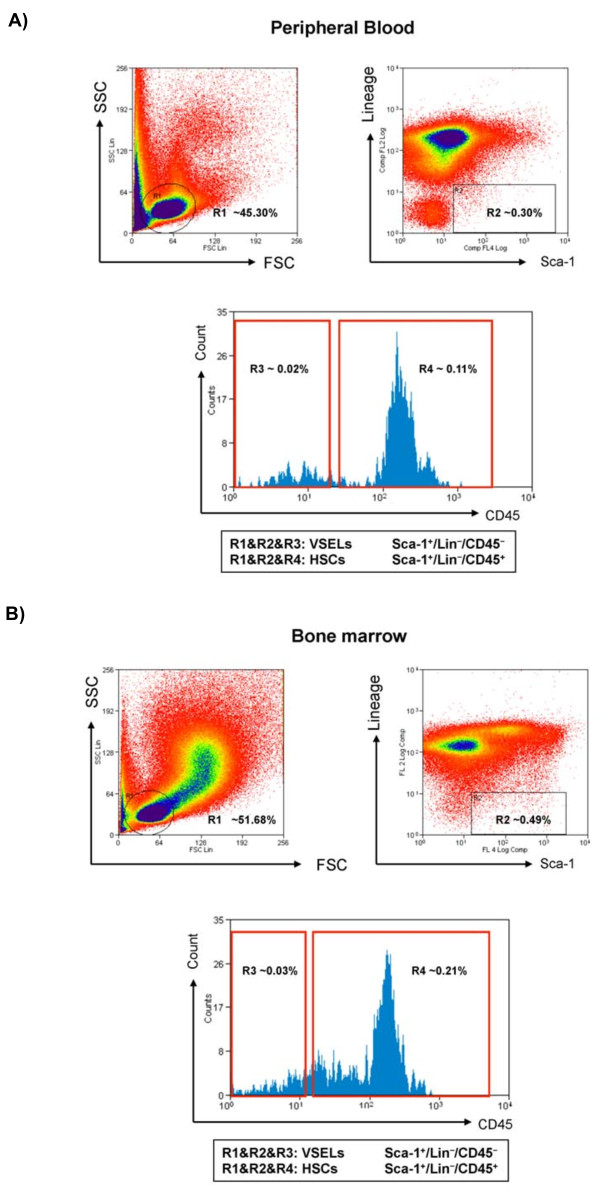
**Isolation of VSELs from peripheral blood (Panel A) and bone marrow (Panel B) using FACS**. Forward scatter (FSC) and side scatter (SSC) distribution of beads with a range of diameters (1-15 μm, not shown) identified region 1 (R1) and was used to select populations of peripheral blood (Panel A) or bone marrow mononuclear cells (Panel B) ranging in size between 2-10 μm (R1). The small, low complexity cells in R1 were further separated using expression of Sca-1 and Lineage markers (R2: Sca-1^+ ^Lin^-^), followed by sorting using CD45 marker expression into two distinct populations (R3, R4): hematopoietic stem cells (HCS, Sca-1^+ ^Lin^- ^CD45^+^), and very small embryonic-like stem cells (VSELs, Sca-1^+ ^Lin^- ^CD45).

VSELs were isolated from murine bone marrow and peripheral blood nucleated cells using multicolor fluorescence-activated cell sorting (FACS) as previously described [4,12,23]. Briefly, the population of total nucleated cells (TNCs) from murine PB and BM was obtained after lysis of red blood cells using 1× BD Pharm Lyse Buffer (BD Pharmingen, San Jose, CA, USA). TNCs were subsequently stained for hematopoietic lineage markers (Lineage - lin), for Sca-1 and CD45 antigens, for 30 minutes in medium containing 2% fetal bovine serum. The following anti-mouse antibodies (BD Pharmingen, San Jose, CA, USA) were used for staining: anti-CD45R/B220 (phycoerythrin - PE; clone RA3-6B2), anti-Gr-1 (PE; clone RB6-8C5), anti-T-cell receptor-αβ (PE; clone H57-597), anti-T-cell receptor-γδ (PE; clone GL3), anti-CD11b (PE; clone M1/70), anti-Ter119 (PE; clone TER-119), and anti-Ly-6A/E (Sca-1) (biotin; clone E13-161.7, with streptavidin-conjugated to PE-Cy5) and rat anti-CD45 (allophycocyanin-Cy7; clone 30-F11). All mAbs were added at saturating concentrations. Cells were then washed, re-suspended in RPMI 1640 medium with 10% fetal bovine serum, and sorted by MoFlo cell sorter (Dako, Carpinteria, CA, USA). The Sca-1+Lin-CD45- cells (VSELs) and control Sca-1+Lin-CD45+ cells (HSCs) were isolated according to the gating and sorting strategy described before and outlined in Figure 1. For each sample run, at least 10^6 ^events were acquired, with VSELs accounting for 0.02-0.03% of the total cells (BM or PB). Given the scarcity of VSELs in peripheral blood and bone marrow, we performed six independent measurements per exposure condition (hypoxia, normoxia), each based on pooled samples from 10 mice, for a total of 120 animals.

### Measurement of plasma chemokines

Mouse blood was collected from the vena cava in vacutainer tubes containing EDTA (Becton Dickinson, Franklin Lakes, NJ, USA). The collected fresh blood was centrifuged at 2000 × g for 20 min at 4°C, subsequently plasma was centrifuged for 5 minutes at 13,000 rpm to remove remaining cells and platelets, and immediately frozen at -80°C until further analysis. Levels of stem cell chemokines stromal cell derived factor-1 (SDF-1), hepatocyte growth factor (HGF), and leukemia inhibitory factor (LIF) were measured with ELISA kits (R&D Systems, Minneapolis, MN) [23]. For the hypoxic exposure, 20 independent measurements and for the normoxic exposure, 30 independent measurements were performed. For each of these measurements, plasma was pooled from 3-4 mice.

### Statistical analysis

Chemokine levels and VSEL counts between hypoxia and normoxia were performed using unpaired, two-tailed Student's *t*-test with *P*-values adjusted for unequal variances when appropriate. Box and whisker representation of the data was used to depict the upper and lower quartiles (box) and the maximum and minimum values (whiskers).

### Microarray experiments

VSELs were isolated from the bone marrows of 30 additional mice exposed to hypoxia and 30 mice exposed to room air (pooled into six groups of n = 10). Total RNA was isolated using PicoPure RNA Isolation Kit (Arcturus Bioscience Inc., Mountain View, CA) and amplified using Low-RNA Input Fluorescent Linear Amplification kit (Agilent Technologies, Santa Clara, CA) with modifications. For each of the pooled groups (n = 10 mice per group), labeled cRNA was hybridized to an Agilent murine whole-genome 60-mer oligo microarray (Agilent Technologies, Santa Clara, CA) comprised of over 45,000 probes as previously described [23]. Six independent hybridizations-3 pooled samples from mice exposed to hypoxia and 3 pooled samples from normoxic mice, were performed. After image processing, background-subtracted intensities were normalized using the quantile method [24].

### Microarray data analysis

Two-dimensional hierarchal clustering using Pearson's correlation was performed on the entire normalized gene expression data [25]. Differentially expressed genes in VSELs exposed to hypoxia versus normoxia were identified using a Bayesian implementation of the parametric *t*-test [26] and corrected for multiple comparisons with the *Q*-value method [27]. A *Q*-value of < 0.05 was selected for identifying significant differential gene expression.

### Gene ontology analysis

Differentially expressed genes in VSELs (*Q*-value < 0.05) underwent functional analysis with a web-based program, Database for Annotation, Visualization and Integrated Discovery (DAVID) and categorized using the Gene Ontology database [28]. A permutation-based FDR analysis was employed (FDR cutoff < 5%).

### Network analysis

By including all differentially expressed genes during hypoxia as initial seeds, we developed a gene product relational network based on published interaction databases [29-31]. To maximize the accuracy of this "interactome", we limited the connection between any two nodes to known direct relationships (e.g., protein-protein interaction). The topological architecture of the network was derived from its connectivity matrix. We tested the "scale-free" property of the network by determining if it followed a power law distribution [32]. We identified the key network hubs by selecting differentially expressed nodes with at least 10 connections.

## Results

### Effects of *in vivo *exposure to hypoxia on VSEL populations and stem cell chemokines

We utilized a sequential gating strategy using FACS and based on size, granularity and stem cell-specific antibody staining to purify VSELs from PB and BM of mice exposed to hypoxia and normoxic controls (Figure 1). In peripheral blood, the white blood cell counts were 9,980 ± 467/ml in normoxia and 10,503 ± 420/ml in hypoxia (p > 0.05); a minimum of 100,000 cells were counted from merging the samples from 10 mice to determine the frequency of VSEL in peripheral blood for each independent sample run. For bone marrow, cellularity was estimated from the mixing of 10 mice to include approximately 1 million cells for each independent sample run. Given the scarcity of VSELs in peripheral blood and bone marrow, we performed six independent measurements per exposure condition (hypoxia, normoxia), each based on pooled samples from 10 mice, for a total of 120 animals.

**Figure 2:**
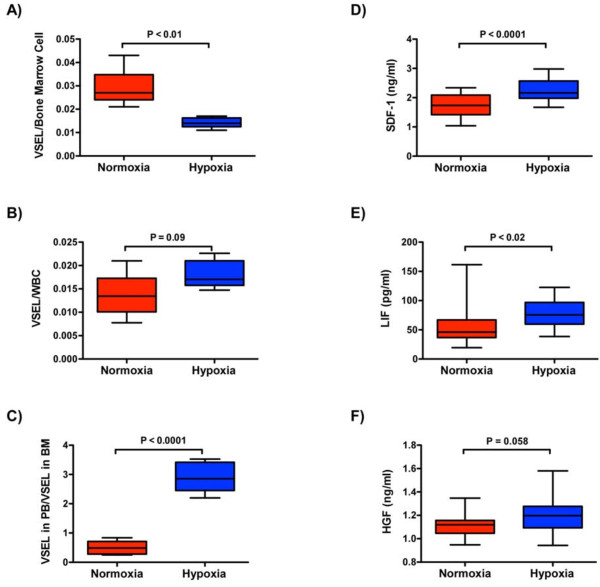
**In response to *in vivo *exposure to hypoxia, VSELs are mobilized from bone marrow (BM) to peripheral blood (PB), and stem cell chemoattractant gradients appear in plasma of mice**. Panels A-C demonstrate that hypoxia causes a significant reduction in BM VSEL number, a concurrent increase in PB VSEL count (although not reaching statistical significance) and an overall significant increase in the relative ratio of these stem cells in PB vs. BM. Panels D-F show increased protein expression of three stem cell chemoattractants in response to hypoxia, stromal cell derived factor-1 (SDF-1), leukemia inhibitory factor (LIF), and hepatocyte growth factor (HGF, does not reach statistical significance). *P*-values were calculated using unpaired Student's *t*-test. Box and whisker plots show the upper and lower quartiles (box) and the maximum and minimum values (whiskers).

Exposure to hypoxia resulted in a significant increase in the peripheral count of VSELs relative to their BM reserve pool (Figure 2A-C). Furthermore, *in vivo *exposure to hypoxia was associated with a significant elevation in plasma levels of the stem cell chemoattractants SDF-1 and LIF, and borderline increase in HGF (Figure 2D-F), providing further evidence that hypoxia promotes a favorable gradient for mobilization of VSELs from BM to PB.

### Transcriptional profiling of VSELs during hypoxia

**Figure 3:**
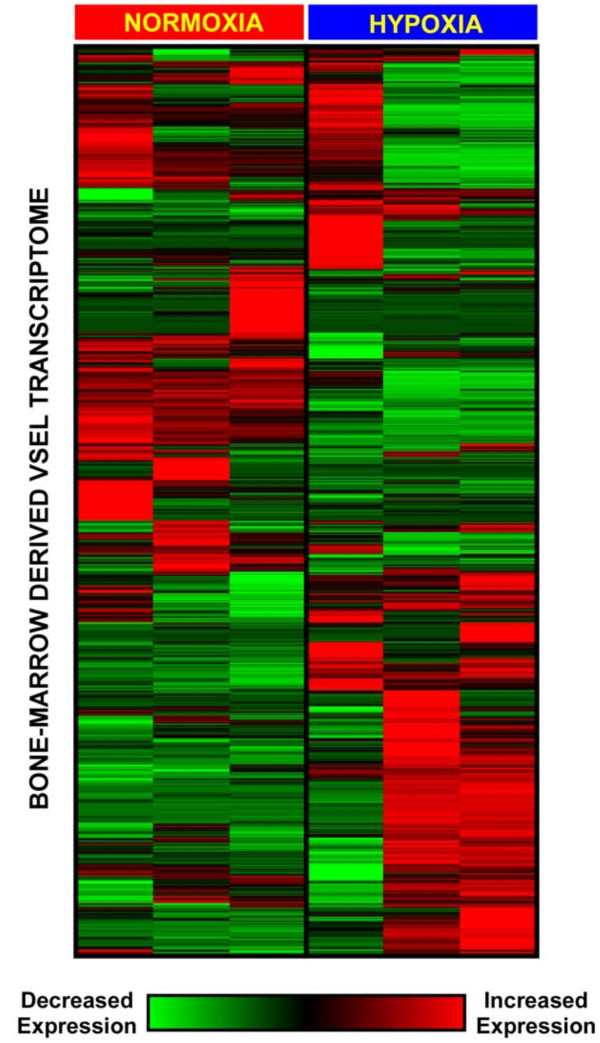
**Two-dimensional hierarchical cluster analysis of bone marrow-derived VSEL gene expression in mice exposed to hypoxia or normoxia**. The distinct gene expression patterns during hypoxic exposure imply genome-wide transcriptional perturbation in these pluripotent stem cells.

Global patterns in gene expression of BM-derived VSELs exposed to hypoxia and normoxic controls were dissected using hierarchical cluster analysis and displayed as a heatmap (Figure 3). This analysis revealed that exposure to hypoxia causes a perturbation in the entire VSEL transcriptome that is distinct from the normoxic condition. Within this genome-wide expression pattern, we identified 1,388 differentially expressed genes using a *Q*-value cutoff of < 0.05 (Additional file 1, Table S1). The subsequent pathway and network analyses were limited to this subset of statistically significant differentially expressed genes.

### Functional and network analyses of hypoxia-induced transcriptional programs in VSELs

**Figure 4:**
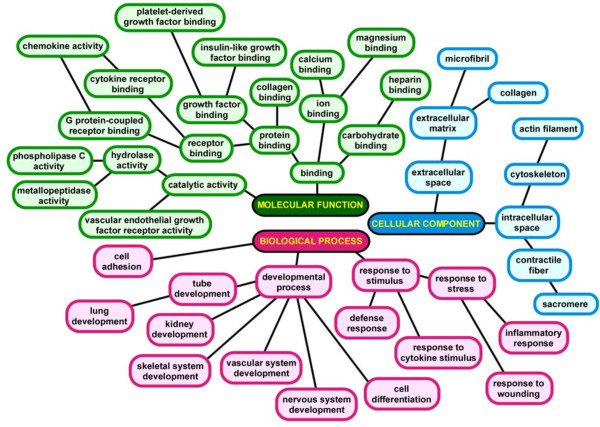
**Functional analysis of enriched categories in VSELs during hypoxic exposure using Gene Ontology's hierarchical annotation tree structure**. Functional categories reaching statistically significant enrichment (FDR cutoff < 0.05) and representing biological processes, molecular functions and cellular components are depicted.

Functional enrichment analysis of differentially expressed genes was performed using Gene Ontology analysis [28] and graphically displayed based the hierarchical structure of this annotation database (Figure 4). *In vivo *exposure to hypoxia activated a diverse array of transcriptional programs in pluripotent VSELs ranging from a number of organ-specific developmental processes (e.g., lung, kidney, skeletal, vascular, and nervous system development) to pathways involved in defense and stress response, cytokine and growth factor binding, and catalytic activity. These results provide a blueprint mapping the profound and widespread functional activation of BM-derived VSELs in response to a brief *in vivo *hypoxic exposure.

**Figure 5:**
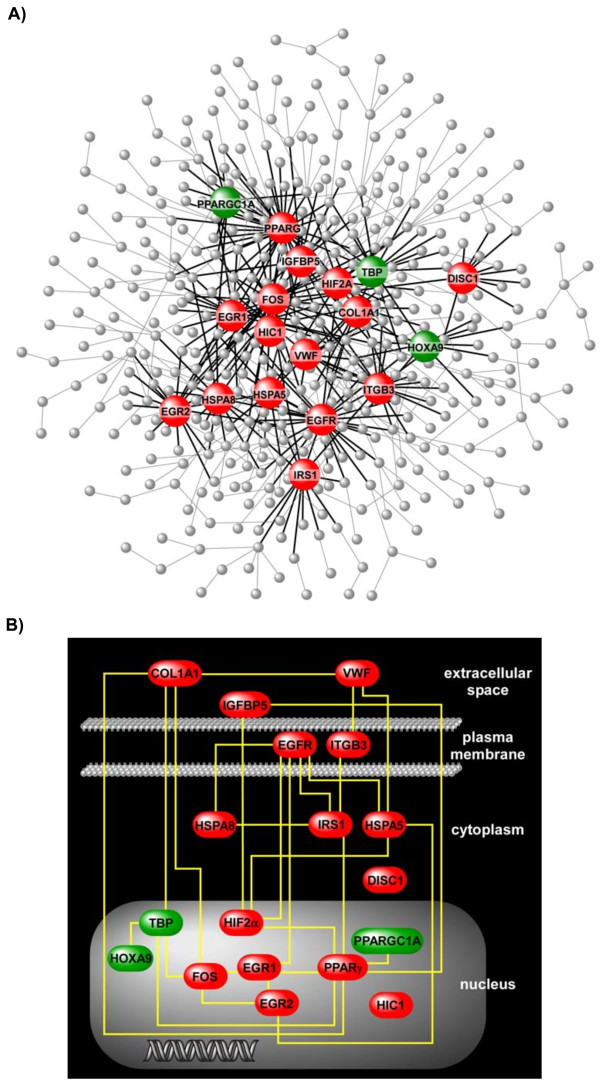
**Differentially expressed hubs of the hypoxia-induced VSEL interactome**. Panel (A) depicts the full network while highlighting the18 hubs with ≥ 10 edges and their respective connections (see also Additional file 2, Figure S1). Note that these densely connected nodes capture a significant proportion of the overall network connectivity (313 out of 604 connections). Red: upregulated in hypoxia, Green: downregulated in hypoxia. Panel (B) is a wiring diagram limited to the interconnections among the network hubs, with each gene product placed within its cellular compartment. This genetic circuit is postulated to encompass key controllers of stem cell responses during *in vivo *hypoxia.

Given the vast transcriptional effects of hypoxia on VSELs, it is highly unlikely that a single gene will solely orchestrate this response. To better elucidate the relationships among differentially expressed genes mapped to the enriched functional groups and identify the key controllers of hypoxia-induced transcriptional programs in VSELs, we developed a gene product interaction network, or interactome, based on previously published direct interactions (Additional file 2, Figure S1). This network consisted of 424 genes (nodes) and 604 connections (edges). Topologically, the interactome possessed scale-free properties [32] because its degree distribution (number of nodes with a given connection, *N_k_*) and nodal connectivity (*k*) were linked via a power law relationship-i.e., *N_k _~ k^-γ ^*(*γ *= 1.64, *R^2 ^*= 0.98) (Additional file 2, Figure S1). We and others have demonstrated that the functional stability of complex biological networks is critically dependent on highly connected nodes or "hubs" [33,34]. We identified the VSEL network hubs by locating 18 differentially expressed nodes with at least 10 interactions (Table 1). As shown in Figure 5A, the connectivity pattern of these hubs captures a significant proportion of the overall network structure (313 edges out of a total of 604). Furthermore, the key nodes themselves are highly interconnected implying coordinated, direct functional relationships between these hubs (Figure 5B).

**Table 1:** Differentially expressed network hubs in VSELs exposed to *in vivo *hypoxia

Node Name	Symbol	Connectivity	Q-value
FBJ osteosarcoma oncogene	*Fos*	42	1.9 × 10^-2^

Peroxisome proliferator activated receptor gamma	*Pparγ*	39	3.9 × 10^-2^

Epidermal growth factor receptor	*Egfr*	28	5.3 × 10^-4^

Early growth response 1	*Egr1*	19	6.9 × 10^-3^

Early growth response 2	*Egr2*	19	2.8 × 10^-2^

Integrin beta 3	*Itgb3*	18	3.3 × 10^-3^

TATA box binding protein	*Tbp*	15	2.2 × 10^-2^

Hypoxia-inducible factor 2 alpha	*Hif2α*	15	1.7 × 10^-2^

Collagen type I alpha 1	*Col1a1*	15	2.5 × 10^-2^

Heat shock protein 8	*Hspa8*	14	4.1 × 10^-2^

Insulin receptor substrate 1	*Irs1*	13	8.9 × 10^-5^

Insulin-like growth factor binding protein 5	*Igfbp5*	12	5.7 × 10^-7^

Heat shock protein 5	*Hspa5*	11	2.5 × 10^-2^

Disrupted in schizophrenia 1	*Disc1*	11	8.1 × 10^-3^

Homeobox A9	*Hoxa9*	11	1.8 × 10^-2^

Von Willebrand factor homolog	*Vwf*	11	9.1 × 10^-3^

Peroxisome proliferative activated receptor gamma coactivator 1 alpha	*Ppargc1a*	10	2.1 × 10^-4^

Hypermethylated in cancer 1	*Hic1*	10	2.6 × 10^-2^

## Discussion

The main finding of this work is that exposure to hypoxia-a common characteristic of many lung disorders, recruits pluripotent VSELs from BM to the peripheral blood and induces a distinct gene expression signature in these stem cells. Using a computationally-intensive approach, we mapped the functional and network properties of activated transcriptional programs and identified hubs that may serve as key regulators of the VSEL response to hypoxia.

To our knowledge, this is the first report on the global transcriptional effects of hypoxia on stem cells using *in vivo *exposure at physiologically relevant oxygen partial pressures. Utilizing a similar strategy, we recently used a murine model to investigate the consequences of cyclical hypoxia during sleep on BM-derived VSELs [23]. The intermittent hypoxic exposure was intended to model obstructive sleep apnea and that project was therefore more narrowly defined in its focus and clinical relevance. Nevertheless, several of our current findings were also observed in the intermittent hypoxia system, including mobilization of VSELs from BM to PB, elevation of stem cell chemoattractant chemokines, and enrichment of transcriptional programs involved in development and organogenesis. Network analysis of the differentially expressed genes in VSELs also revealed that some of the key hubs were shared between the hypoxia models (intermittent, sustained), including hypoxia inducible factor 2 alpha (*Hif2α*), proliferator-activated receptor gamma (*Pparγ*), integrin beta 3 (*Itgb3*), and insulin growth factor binding protein 5 (*Igfbp5*). This observation implies that global exposure to reduced oxygen, whether continuous or intermittent, activates a number of common pathways in pluripotent stem cells. However, there were also significant differences in the transcriptional programs enriched in VSELs under sustained vs. intermittent hypoxic exposure. Firstly, a larger number of genes were differentially expressed during continuous hypoxia suggesting a more profound perturbation. Secondly, a much broader spectrum of pathways was enriched in sustained compared to intermittent hypoxia that extended beyond developmental programs and included processes involved in defense and stress response, wound healing, chemokine activity, cell adhesion and structure (Figure 4). Thirdly, many of the network hubs identified during exposure to continuous hypoxia were not differentially expressed during cyclical hypoxia (Figure 5, Table 1), including the most densely connected node, *Fos *(FBJ osteosarcoma oncogene), and several other key transcriptional regulators such as early growth response 1 and 2 (*Egr1, Egr2*), and TATA box binding protein (*Tbp*).

*Fos *and *Egr1 *are prototypic master regulators known as immediate early-genes (IEG) that orchestrate widespread activation of cellular gene expression in response to specific perturbations, including hypoxia, ischemia, atherosclerosis, angiogenesis, and neuronal survival [35-37]. *Egr2 *has been shown to govern the development and segmentation of the mouse hindbrain [38], and mutations in this gene have been associated with congenital hypomyelinating neuropathies in humans [39]. Intriguingly, a recent study demonstrated that a population of *Egr2*-expressing precursor cells develop into the first embryonic respiratory rhythm-generating neuronal circuit [40], and *Egr2^-/- ^*transgenic mice die from breathing irregularities resulting in severe hypoxia [41].

Two members of the heat shock protein 70 group, *Hspa8 *and *Hspa5*, were also densely connected network nodes whose expression increased significantly during hypoxia. Products of these genes play crucial roles in protecting cells from environmental stressors including high temperature, oxidative stress, ischemia and hypoxia [42]. More specifically, targeting *Hspa8 *and *Hspa5 *has been demonstrated to protect cardiomyocytes [43] and mesenchymal stem cells from hypoxia-induced apoptosis [44], whereas delivering these heat shock proteins into transplanted mesenchymal stem cells rescues heart function after myocardial infarction in rats [44].

Another differentially upregulated hub within the interactome, *Hif2α*, is a member of the HIF family of basic helix-loop-helix transcription factors that control the global cellular response to oxygen deprivation. *Hif2α *overexpression is seen many solid tumors characterized with significant hypoxic burden including renal cell carcinoma, non small cell lung cancer and meduloblastoma, and is associated with poor outcome in these cancers [45]. Recent reports suggest that *Hif2α *is a critical activator of a subpopulation of cancer cells with stem cell-like properties [46,47], and pharmacologic and genetic targeting of this gene affects differentiation of progenitor stem cells [48,49]. Interestingly, a single nucleotide polymorphism in *Hif2α *was recently demonstrated to be highly associated with adaptability to high altitude hypoxia in ethnic Tibetans [50].

The mobilization of VSELs from BM to peripheral circulation in response to oxygen deprivation implies the activation of organ-specific transcriptional programs under regulatory controls. Our network analysis provided an overview of the transcriptional landscape of these pluripotent stem cells during hypoxia. Since the functional integrity of such scale-free biologic networks is dependent on densely connected nodes [33], we postulate that these hubs comprise key regulators of hypoxia-induced gene expression in VSELs. As depicted in Figure 5, the hubs not only form the structural foundation of the interactome, but are themselves highly interconnected via direct interactions, implying a high degree of functional coordination in orchestrating the hypoxia-induced transcriptional response of stem cells.

To explore whether this hypoxia-induced transcriptional signature is unique to VSELs or common among leukocyte subpopulations, we compared our results to expression profiles of mononuclear cells (MNCs) and mesenchymal stem cells (MSCs) under hypoxia as reported by Onishi *et al *[51]. While there are substantial differences between the two studies in terms of experimental protocols, hypoxic exposures, and microarray platforms, less than 1% of the differentially expressed genes in VSELs were significantly altered in MSCs or MNCs, suggesting that many of the hypoxia-induced genes in VSELs are exclusive to this subpopulation of cells. However, from a functional standpoint, differentially expressed genes in VSELs and MSCs were both enriched in several developmental processes, whereas MNCs were highly enriched in defense/immune responses and inflammation. Therefore, VSELs appear to possess transcriptional programs in common with both MSCs and MNCs-a finding that is consistent with their pluripotent properties.

Several limitations in the current study deserve mention. We have only explored the effects of hypoxia on gene expression and not assessed the post-transcriptional modifications of gene products activated during hypoxic exposure. The duration of hypoxia was short-the long term consequences of chronic oxygen deprivation on VSELs were not investigated in this study and represent a future research goal. Although bone marrow is the primary depot of VSELs, our results do not preclude the possibility that some of these stem cells were also mobilized from other tissue niches during exposure to hypoxia. Nevertheless, our expression profiling experiments were focused on BM-derived VSELs. We have not identified the tissue source of hypoxia-induced elevation in stem cells chemokines, but given the extremely low number of circulating VSELs, we believe it is unlikely that VSELs are the primary source. Although the bone marrow is a known source of these chemoattractants, hypoxic injury to various tissues can lead to the release of the chemokines into peripheral blood [5,14,16]. Finally, we did not follow the fate of mobilized VSELs to identify their target end organs, although given the global effects of hypoxia and the diversity of enriched developmental programs, we expect that these stem cells will be recruited to multiple sites and undergo tissue-specific differentiation.

## Conclusions

Collectively, our results demonstrate that *in vivo *exposure to hypoxia in mice elicits chemoattractant gradients that promote the mobilization of pluripotent very small embryonic-like stem cells from the bone marrow to peripheral blood. A computationally-driven analysis of the transcriptome of these cells highlighted the activation of diverse transcriptional programs that appeared to be coordinated by a select number of highly connected network nodes. These hubs may represent critical targets for modulating the functional consequences of hypoxic burden on stem cells. Our approach provides a general framework for the systematic study of stem cell biology under clinically relevant pathophysiologic perturbations.

## Competing interests

The authors declare that they have no competing interests.

## Authors' contributions

SAG analyzed the data and wrote the manuscript. DG conceived the study, participated in its design and draft. AK participated in designing the study and collected the stem cell data and performed the transcriptional profiling experiments. MK assisted with FACS-based stem cell isolation and collection of stem cell data. EAD, JK, and HBC assisted with the stem cell isolation, animal experiments, and stem cell data measurements. All authors read and approved the final manuscript.

## Supplementary Material

Additional file 1 **Tabular list of differentially expressed genes in VSELs after hypoxic exposure**. This file contains the list of differentially expressed genes (based on a Q-value cutoff less than 0.05) in VSELs exposed to hypoxia. The list includes the official gene symbols, Entrez Gene IDs, Q-values, and Log_2_[expression in hypoxia/expression in normoxia]. Click here for file

Additional file 2 **Gene product interaction network of differentially expressed genes in VSELs**. This file contains two figures depicting a gene product interaction network of differentially expressed genes in VSELs after *in vivo *exposure to hypoxia. Panel (A) highlights up and downregulated genes (red and green respectively). This network has 424 genes (nodes) and 604 connections (edges). Note that each connection represents a known direct interaction between two gene products. Panel (B) demonstrates that the topology of this network is scale-free because it follows a power law distribution. *N*_*k*_, degree distribution; *k*, nodal connectivity. Click here for file
