# Supplementary material for: Transcriptional landscape of bone marrow-derived very small embryonic-like stem cells during hypoxia
Source: Respir Res. 2011 May 10;12(1):63. doi: 10.1186/1465-9921-12-63 (PMC3098802; doi:10.1186/1465-9921-12-63)
Supplement: Additional file 1 — Tabular list of differentially expressed genes in VSELs after hypoxic exposure. This file contains the list of differentially expressed genes (based on a Q-value cutoff less than 0.05) in VSELs exposed to hypoxia. The list includes the official gene symbols, Entrez Gene IDs, Q-values, and Log2[expression in hypoxia/expression in normoxia]. [file 1465-9921-12-63-S1.PDF]

Supplementary Table 1. List of differentially expressed genes in VSELs after hypoxic exposure. False discovery rate analysis using Q-value < 0.05 was used to designate differential expression.

Gene Symbol	Entrez Gene ID	Q-value	Log_2[expression ratio]
Aanat	11298	9.01E-07	-3.168
Abca2	11305	3.59E-02	-1.814
Adipoq	11450	8.44E-07	3.757
Ada	11486	3.68E-02	-0.593
Adam19	11492	6.71E-03	3.583
Adamts1	11504	8.67E-06	3.653
Adcy6	11512	1.38E-03	1.567
Adcyap1r1	11517	3.34E-03	2.617
Adora1	11539	1.81E-06	-2.737
Adra2a	11551	1.11E-02	1.864
Adssl1	11565	5.82E-03	-0.580
Afp	11576	7.09E-03	-1.417
Angpt2	11601	2.77E-02	1.674
Agt	11606	3.63E-04	2.182
Alas2	11656	2.61E-03	1.165
Alox12	11684	1.40E-02	3.412
Ambp	11699	3.21E-03	1.852
Amy1	11722	2.64E-04	2.124
Ap1g1	11765	1.56E-03	1.309
Ap1s1	11769	2.96E-03	-0.821
Fabp4	11770	4.54E-02	1.213
Ap3b2	11775	2.94E-03	-2.779
Ap4m1	11781	1.09E-02	-1.619
Speg	11790	9.30E-03	2.561
Cd5l	11801	7.96E-04	-1.429
Aplp2	11804	1.68E-02	0.781
Apoe	11816	8.55E-05	1.053
Rplp0	11837	3.87E-04	0.956
Arf1	11840	3.37E-02	0.773
Arl4a	11861	1.81E-03	2.768
Arvcf	11877	3.68E-07	-3.065
Atp1a1	11928	1.32E-02	0.573
Atp4a	11944	3.96E-02	-1.599
Atp6v0a1	11975	2.39E-02	0.784
Bmp7	12162	2.23E-02	1.328
Serping1	12258	1.27E-05	3.872
C2	12263	1.10E-02	2.011
C4b	12268	4.86E-05	3.229
Cacna2d1	12293	4.36E-03	3.107
Cacng1	12299	1.34E-02	3.055
Calb1	12307	2.10E-07	-2.592
Calca	12310	3.82E-02	-0.702
Calm2	12314	2.25E-02	0.461
Calr	12317	4.52E-02	-0.592
Capzb	12345	1.13E-02	-2.155

Car1	12346	3.57E-04	1.161
Car2	12349	3.92E-03	1.174
Casp12	12364	4.87E-07	4.641
Casp7	12369	2.99E-02	-1.780
Cast	12380	4.83E-02	-1.031
Cbfa2t2	12396	1.49E-02	-2.280
Cbfa2t3	12398	3.41E-02	-0.711
Cbln1	12404	6.57E-06	4.356
Rb1cc1	12421	4.43E-02	0.973
Ccnk	12454	4.02E-03	-1.930
Cd34	12490	6.06E-03	-1.006
Cd37	12493	4.99E-02	-1.095
Cd5	12507	1.80E-02	-1.060
Cd6	12511	2.03E-02	-0.987
Cd80	12519	4.22E-02	-1.219
Cd8b1	12526	4.19E-03	-1.167
Cdc25b	12531	1.31E-03	-2.206
Cdk6	12571	1.95E-02	-1.832
Cdyl	12593	3.23E-02	0.861
Cenpb	12616	4.56E-03	-1.804
Cfh	12628	3.58E-04	1.756
Chga	12652	9.94E-05	-3.308
Chgb	12653	3.70E-03	-2.338
Socs3	12702	2.90E-02	0.775
Clca1	12722	1.38E-02	2.116
Clcn5	12728	1.85E-02	2.577
Clk1	12747	7.85E-03	0.591
Clu	12759	4.24E-04	2.846
Cxcr7	12778	1.63E-04	3.079
Cnga2	12789	2.13E-02	1.893
Cnp	12799	4.24E-03	-1.265
Cntn1	12805	8.80E-03	-3.022
Cobl	12808	1.51E-02	2.111
Col11a1	12814	2.08E-02	3.036
Col18a1	12822	1.09E-02	2.066
Col3a1	12825	4.94E-04	2.384
Col4a1	12826	1.02E-02	2.270
Col6a1	12833	4.33E-02	2.625
Col8a1	12837	2.78E-05	3.398
Col1a1	12842	2.53E-02	1.793
Col1a2	12843	3.50E-04	3.090
Cox6a2	12862	2.77E-03	-0.891
Cp	12870	1.87E-06	3.949
Cpa3	12873	7.80E-03	-1.197
Cplx2	12890	1.28E-03	-2.052
Cr2	12902	3.82E-02	-1.704
Crcp	12909	4.60E-02	0.828
Crlf1	12931	3.40E-02	1.116
Cry1	12952	4.50E-03	3.544

Csf1r	12978	2.89E-02	-1.406
Csf2	12981	7.93E-03	-1.749
Dnajc5	13002	3.22E-02	0.943
Ncan	13004	1.22E-02	-1.870
Cst7	13011	2.74E-02	-0.691
Ctf1	13019	4.26E-02	-1.793
Ctla2a	13024	2.54E-03	1.168
Ctla2b	13025	9.93E-03	1.221
Ctsg	13035	3.63E-05	-1.054
Ctsl	13039	1.87E-02	0.692
Cyct	13067	1.20E-02	2.827
Cyp1b1	13078	5.03E-05	5.315
Dach1	13134	3.06E-02	-1.335
Dapk3	13144	4.22E-03	-2.289
Dcn	13179	2.65E-07	5.134
Asap1	13196	3.07E-03	0.933
Twist2	13345	3.57E-04	2.774
Dgat1	13350	1.95E-02	-0.896
Dmp1	13406	1.69E-07	5.518
Dpep1	13479	4.40E-05	4.004
Drg2	13495	4.49E-02	-1.093
Dst	13518	3.54E-02	2.273
Dyrk1a	13548	4.97E-02	-1.109
Ebf1	13591	4.47E-03	-1.821
Ebf3	13593	2.16E-04	3.267
Ecm1	13601	2.72E-03	1.312
S1pr3	13610	2.13E-04	2.705
Edn1	13614	3.43E-03	2.847
Ednra	13617	1.51E-02	1.637
Efna5	13640	1.26E-03	2.520
Efnb1	13641	3.57E-02	0.853
Efnb3	13643	8.02E-06	-2.470
Efs	13644	4.28E-03	3.561
Egfr	13649	5.29E-04	2.145
Egr1	13653	6.93E-03	2.027
Egr2	13654	2.82E-02	0.986
Egr4	13656	4.24E-03	-2.416
Emp2	13731	9.88E-03	2.773
Emr1	13733	2.98E-02	3.215
Hif2a	13819	1.70E-02	1.685
Erg	13876	1.06E-02	1.033
Gnb1l	13972	1.80E-02	-0.731
Eya4	14051	2.13E-02	2.253
F2r	14062	5.66E-03	1.213
Fabp1	14080	9.33E-03	3.137
Fap	14089	2.03E-02	2.801
Fas	14102	3.11E-02	0.987
Fat1	14107	3.82E-04	3.667
Fbln2	14115	7.85E-03	-2.606

Fbn1	14118	1.44E-02	3.214
Fbn2	14119	1.44E-04	3.619
Fcnb	14134	2.26E-02	-0.700
Fdft1	14137	2.86E-02	-1.246
Lgr5	14160	2.53E-02	-1.297
Fgf12	14167	8.47E-03	-1.969
Fgf7	14178	1.14E-02	2.626
Fgfr1	14182	1.96E-02	-1.489
Fhl1	14199	2.10E-02	3.260
Fhl2	14200	6.28E-03	1.882
Ctgf	14219	4.71E-06	3.156
Fkbp10	14230	2.89E-02	2.274
Fkbp7	14231	4.98E-03	1.297
Fos	14281	1.89E-02	1.284
Fosl2	14284	7.64E-03	-1.573
Cidec	14311	2.22E-02	-1.975
Fstl1	14314	1.24E-05	3.500
Fzd1	14362	1.43E-03	2.204
Gad1	14415	7.14E-03	-1.939
Galc	14420	3.25E-02	-1.528
Gas6	14456	2.41E-05	2.621
Arhgdig	14570	2.17E-02	1.752
Gfpt1	14583	3.49E-02	0.776
Gfra1	14585	8.12E-03	2.404
Gfra3	14587	3.61E-02	1.920
Ghr	14600	4.33E-04	3.580
Ghrh	14601	2.39E-02	-1.665
Gja1	14609	1.98E-04	3.535
Gjc1	14615	2.13E-04	2.813
Gjb2	14619	2.22E-06	-2.701
Gli3	14634	3.40E-03	2.491
Glud1	14661	2.11E-02	0.462
Gna11	14672	9.11E-03	-1.559
Gna13	14674	4.70E-02	-1.692
Gnai2	14678	5.42E-03	-0.796
Gnaz	14687	1.20E-02	4.387
Gng8	14709	4.83E-02	-1.719
Gp1ba	14723	3.04E-03	5.211
Gp1bb	14724	4.57E-02	1.577
Pdpn	14726	4.91E-02	2.764
Gpr162	14788	2.19E-03	-1.531
Aes	14797	2.89E-02	-0.956
Cxcl1	14825	1.42E-03	3.171
Hspa5	14828	2.51E-02	0.563
Gstm1	14862	4.61E-02	-1.024
Gstm2	14863	4.96E-02	1.185
Gstt2	14872	4.60E-02	-0.856
Gtf2i	14886	1.08E-02	-1.301
Trip12	14897	3.49E-02	1.019

Gtpbp1	14904	1.57E-02	-2.081
Guca2b	14916	3.30E-02	-2.350
Gzmb	14939	1.59E-02	-1.458
H2-Aa	14960	3.92E-02	-1.054
Cfb	14962	1.40E-02	1.514
H2-DMb1	14999	2.01E-02	-0.694
Mr1	15064	7.06E-03	-1.867
Hadh	15107	2.67E-02	-1.268
Hap1	15114	2.91E-02	-1.292
Hba-a1	15122	2.66E-02	0.787
Hbb-b1	15129	1.09E-07	1.320
Hcfc1	15161	3.54E-02	1.436
Hbegf	15200	2.01E-02	1.149
Foxg1	15228	3.97E-03	-2.440
Mst1	15235	6.67E-03	-2.197
Hhex	15242	7.83E-03	-1.536
Hic1	15248	2.58E-02	1.657
Hipk2	15258	2.90E-02	-2.110
Hipk3	15259	7.06E-03	-2.333
H2afx	15270	3.19E-02	-0.564
Hk1	15275	1.17E-04	-2.620
Hlx	15284	1.06E-02	-1.136
Hmbs	15288	3.92E-02	0.462
Hmga2-ps1	15365	1.34E-04	-2.602
Hoxa9	15405	1.78E-02	-1.816
Hoxb9	15417	1.19E-02	3.289
Hpx	15458	7.50E-04	2.392
Hspa8	15481	4.09E-02	0.752
Hsd17b1	15485	3.21E-02	-1.648
Hspb1	15507	5.03E-03	1.986
Sdc2	15529	6.76E-03	2.893
Elavl2	15569	7.14E-03	-2.553
Id4	15904	3.57E-04	2.632
Ifnb1	15977	3.02E-03	-2.635
Ifng	15978	3.18E-02	-0.788
Ifrd1	15982	4.13E-02	-1.188
Ifrd2	15983	4.08E-02	-1.136
Igf2	16002	3.60E-02	2.355
Cyr61	16007	1.36E-06	4.180
Igfbp5	16011	5.74E-07	4.126
Igh-VJ558	16061	2.13E-06	-2.529
Igk-V38	16120	1.37E-04	-2.352
Igll1	16136	3.04E-04	-2.316
Igl-V1	16142	3.91E-05	1.102
Ihh	16147	2.73E-06	-2.802
Il11ra1	16157	1.08E-02	-1.016
Il18rap	16174	3.13E-02	1.046
Il1r1	16177	4.77E-03	1.516
Il1rap	16180	1.35E-02	-3.411

Il2rb	16185	1.34E-02	-1.062
Il6	16193	1.16E-02	-1.151
Il7	16196	3.72E-02	1.913
Ilf3	16201	3.49E-02	1.468
Inhbc	16325	2.31E-02	-3.207
Invs	16348	5.82E-03	-2.001
Irs1	16367	8.87E-05	2.236
Itga2b	16399	3.05E-03	3.845
Itga5	16402	1.17E-02	-2.271
Itgb3	16416	3.34E-03	2.383
Itm2a	16431	9.51E-03	1.218
Jag2	16450	4.54E-02	-2.224
Jup	16480	2.25E-02	2.085
Kcna1	16485	6.64E-03	-1.950
Kcnj10	16513	2.93E-02	-1.239
Kcnj8	16523	2.89E-02	1.470
Kif17	16559	9.06E-04	-2.636
Kif1a	16560	1.68E-03	-1.746
Kif5a	16572	1.69E-03	-1.235
Kifap3	16579	3.93E-02	0.981
Klra5	16636	3.62E-02	-3.472
Kng1	16644	8.91E-06	4.793
Sspn	16651	2.64E-04	3.234
Krt18	16668	4.52E-02	-0.848
Krt86	16679	3.08E-03	-1.537
Krt8	16691	2.69E-02	-1.268
Lama4	16775	4.54E-03	2.782
Lamb2	16779	1.32E-02	2.466
Laptm5	16792	3.49E-02	-0.962
Arhgef2	16800	1.70E-02	-1.990
Lepr	16847	3.03E-04	3.575
Lfng	16848	3.05E-02	-1.815
Lgals1	16852	6.20E-03	-0.508
Lig3	16882	4.70E-02	3.469
Lipe	16890	3.74E-03	-1.247
Lmna	16905	1.48E-04	0.994
Lmnb1	16906	4.98E-02	-0.507
Psmb9	16912	1.40E-02	-0.640
Lox	16948	2.21E-02	1.960
Loxl1	16949	8.12E-03	2.618
Lpl	16956	2.99E-05	1.989
Lrp6	16974	1.52E-02	2.239
Lrp8	16975	2.99E-02	-1.893
Lta	16992	3.39E-02	-1.220
Ltbp2	16997	9.83E-05	2.640
Lum	17022	3.72E-05	3.488
Ly86	17084	4.01E-02	-0.567
Lyz2	17105	4.34E-02	0.560
Marcks	17118	5.82E-03	0.762

Mafg	17134	1.34E-02	1.025
Mfap2	17150	1.50E-04	2.099
Matk	17179	1.77E-02	-1.104
Mbp	17196	2.89E-02	-2.052
Meg3	17263	2.51E-02	2.017
Mest	17294	2.27E-02	2.639
Foxc1	17300	1.74E-04	3.345
Kitl	17311	1.74E-04	3.133
Mgl1	17312	2.43E-02	-0.909
Mgp	17313	9.48E-09	4.639
Mif	17319	3.60E-02	-0.657
Cxcl9	17329	6.93E-03	1.584
Mki67	17345	1.59E-02	0.859
Mme	17380	1.03E-07	4.318
Mmp13	17386	6.71E-05	5.227
Mmp24	17391	3.65E-02	2.098
Mns1	17427	6.41E-03	0.668
Mobp	17433	3.61E-04	-2.433
Mpl	17480	1.58E-02	3.398
Mpo	17523	3.63E-04	-1.002
Mrc2	17534	2.57E-02	2.164
Mre11a	17535	2.60E-02	1.087
Mrvi1	17540	1.64E-02	6.278
COX2	17709	1.95E-03	1.222
Mt2	17750	2.81E-06	1.192
Mapt	17762	2.63E-02	-1.934
Mtm1	17772	1.43E-02	-1.149
Mx1	17857	8.78E-04	-2.251
Myh4	17884	8.84E-03	4.948
Myh8	17885	1.23E-02	5.993
Myl4	17896	6.41E-03	-0.972
Myl1	17901	3.09E-02	3.041
Myo1b	17912	4.32E-02	2.060
Myo6	17920	1.99E-02	1.931
Nbl1	17965	6.32E-03	1.044
Ncam1	17967	4.62E-02	1.274
Ncl	17975	4.83E-03	0.982
Ndufa4	17992	1.68E-02	-0.749
Neo1	18007	4.56E-02	1.397
Nfe2l2	18024	2.77E-02	0.737
Nfib	18028	1.02E-02	1.768
Nefl	18039	2.41E-05	-2.408
Ngfr	18053	4.19E-03	-2.374
Nnmt	18113	2.00E-05	3.993
Nos1	18125	1.66E-02	-1.646
Notch3	18131	2.72E-03	2.271
Nppc	18159	1.48E-06	-2.827
Npr3	18162	4.19E-02	2.929
Nrap	18175	2.01E-02	-2.108

Nrp2	18187	2.16E-02	0.835
Nsg1	18196	2.28E-02	2.083
Nsg2	18197	3.83E-02	-0.972
Nthl1	18207	2.65E-02	-1.936
Ogdh	18293	1.99E-02	-0.933
Ogn	18295	6.62E-05	3.295
Osmr	18414	1.30E-03	2.716
Pafah1b3	18476	2.03E-02	-0.831
Pappa	18491	8.21E-04	3.249
Pcnt	18541	2.85E-02	-1.990
Pcolce	18542	5.14E-05	2.845
Pcsk2	18549	7.43E-06	-2.444
Pcsk5	18552	2.37E-02	2.698
Pcsk6	18553	1.07E-06	5.263
Pdcd1	18566	1.43E-02	-2.821
Pdcd11	18572	4.02E-04	2.431
Pde9a	18585	1.23E-02	2.266
Pdgfra	18595	4.69E-04	3.762
Pdgfrb	18596	1.17E-04	2.574
Padi2	18600	3.60E-02	-0.871
Enpp1	18605	1.82E-02	2.504
Per3	18628	2.04E-02	-1.913
Abcb4	18670	1.32E-02	0.718
Phex	18675	3.96E-02	3.745
Pik3r3	18710	5.91E-03	1.965
Pitx1	18740	2.67E-02	2.459
Pla2r1	18779	9.69E-05	3.463
Pla2g5	18784	3.09E-02	2.004
Plcb2	18796	4.24E-03	-2.668
Plcd1	18799	2.01E-02	1.304
Ccl21a	18829	3.25E-02	5.162
Pmp22	18858	7.20E-05	1.473
Prrx1	18933	5.05E-06	4.195
Pomc	18976	8.80E-03	-1.418
Pou2af1	18985	1.78E-03	-0.704
Pou3f1	18991	2.07E-04	1.622
Pparg	19016	3.92E-02	0.771
Ppargc1a	19017	2.14E-04	-2.734
Prm1	19118	4.89E-03	-2.366
Prps1	19139	3.23E-02	1.134
Lgmn	19141	4.75E-03	0.878
Psap	19156	2.60E-02	-0.855
Ptgfr	19220	8.87E-05	3.803
Ptpn18	19253	1.08E-02	-0.582
Ptprcap	19265	1.04E-02	-0.801
Ptprd	19266	7.38E-04	4.405
Ptrf	19285	1.21E-03	2.016
Ptx3	19288	6.14E-04	2.820
Rab3a	19339	9.30E-03	-1.137

Aldh1a2	19378	4.92E-02	2.563
Robo3	19649	5.63E-04	2.070
Resp18	19711	1.74E-04	-2.872
Rfc2	19718	6.84E-03	-0.699
Rfx1	19724	3.54E-02	2.482
Rfx3	19726	1.95E-02	-1.743
Rgs4	19736	2.39E-02	2.925
Rhag	19743	8.80E-03	2.177
Rmrp	19782	1.69E-03	0.936
Rps12	20042	4.83E-03	0.726
Rps4x	20102	2.94E-02	0.504
Rps7	20115	3.48E-02	0.471
Rxrg	20183	5.68E-04	-2.509
Ncor1	20185	1.74E-02	2.303
S100a11	20195	2.41E-02	-0.773
Saa1	20208	4.26E-02	1.848
Saa2	20209	3.53E-04	2.363
Satb1	20230	4.85E-02	-0.822
Msr1	20288	3.49E-02	1.844
Ccl2	20296	1.74E-02	0.717
Ccl8	20307	1.62E-02	2.357
Cxcl5	20311	2.22E-02	3.698
Cx3cl1	20312	2.34E-02	1.383
Cxcl12	20315	4.39E-07	3.563
Sema3a	20346	4.83E-02	1.902
Sema3e	20349	2.89E-02	3.515
Sfrp1	20377	1.16E-02	2.161
Sfrp4	20379	3.81E-05	2.834
Sftpb	20388	1.38E-03	2.483
Sgce	20392	8.12E-04	2.246
Sgk1	20393	3.92E-03	0.908
Six1	20471	1.26E-03	2.674
Slc16a2	20502	2.67E-02	1.872
Slc19a1	20509	2.70E-02	-1.526
Slc1a1	20510	1.24E-05	-2.619
Slc1a6	20513	1.32E-02	-2.247
Slit2	20563	1.38E-02	3.103
Snai2	20583	8.81E-04	3.466
Smpd2	20598	9.49E-03	-2.085
Siglec1	20612	1.81E-06	-2.965
Snca	20617	1.26E-03	1.271
Son	20658	1.12E-02	0.645
Sox8	20681	7.80E-03	-2.016
Sox9	20682	5.77E-05	3.521
Sparc	20692	1.40E-02	1.420
Sphk1	20698	2.90E-02	0.646
Serpina3g	20715	1.95E-02	0.962
Serpina3n	20716	2.23E-02	2.973
Serpina3m	20717	1.96E-02	1.432

Spint1	20732	5.24E-03	-1.925
Spna2	20740	1.82E-02	-1.226
Spnb1	20741	1.78E-02	2.716
Spp1	20750	8.64E-05	1.532
Stat2	20847	1.75E-02	-1.013
Stim1	20866	2.71E-02	-0.670
Slk	20874	5.56E-03	1.528
Sult1a1	20887	4.28E-02	1.161
Abcc9	20928	3.47E-02	2.178
Surf2	20931	6.67E-03	1.010
Sdc1	20969	2.94E-02	0.981
Tbp	21374	2.22E-02	-0.023
Tbrg4	21379	3.75E-02	-0.668
Tbxa2r	21390	3.48E-02	1.384
Tcf7	21414	4.92E-02	-1.153
Prdx2	21672	1.41E-02	0.492
Dntt	21673	1.45E-02	-0.990
Tead4	21679	3.41E-02	2.995
Terf2	21750	3.12E-02	-0.986
Cirh1a	21771	2.50E-02	1.219
Tgfb1i1	21804	4.21E-02	2.448
Tgfb3	21809	1.05E-03	3.120
Thbs2	21826	1.43E-03	3.420
Thbs3	21827	2.96E-03	2.320
Thy1	21838	9.22E-03	-0.987
Timeless	21853	2.09E-02	-0.861
Timp1	21857	8.28E-04	2.612
Tk1	21877	3.81E-02	-0.754
Tle2	21886	1.37E-03	2.626
Tnnc2	21925	1.01E-02	2.101
Tpbg	21983	1.39E-02	2.197
Tpm1	22003	2.25E-03	1.468
Tpm2	22004	6.79E-03	3.002
Hsp90b1	22027	3.82E-02	-0.538
Traf3	22031	4.49E-02	0.718
Tyro3	22174	8.66E-03	3.562
Dpysl3	22240	7.09E-03	3.426
Upk1b	22268	1.81E-03	2.505
Vegfc	22341	2.64E-04	2.619
Slc32a1	22348	2.96E-03	-1.826
Nrsn1	22360	3.86E-03	-1.431
Vpreb1	22362	2.13E-06	-1.535
Vpreb3	22364	1.48E-03	-0.846
Vwf	22371	9.11E-03	3.156
Wisp1	22402	3.66E-08	4.194
Wisp2	22403	7.80E-05	3.669
Wnt4	22417	2.80E-02	2.207
Siae	22619	1.60E-02	-1.329
Plagl1	22634	3.40E-03	1.941

Zfp13	22654	8.12E-03	2.464
Zfp161	22666	2.89E-02	1.172
Zfp2	22678	2.70E-02	-1.647
Zfp26	22688	9.33E-03	2.438
Zfp27	22689	1.53E-03	2.629
Zfp40	22700	4.80E-02	1.801
Zic1	22771	6.83E-04	-2.363
Adamts5	23794	7.64E-03	1.641
Fbln5	23876	1.42E-03	2.767
Grem1	23892	1.14E-05	3.565
Hs2st1	23908	2.97E-02	-1.668
Mid2	23947	1.64E-02	1.694
Mmp17	23948	9.31E-05	-2.333
Odz4	23966	5.89E-03	3.161
Ccl19	24047	5.97E-04	2.890
2-Sep	24050	2.99E-05	-2.466
Spry1	24063	1.38E-02	1.730
Tlr2	24088	2.96E-02	0.761
Zfp68	24135	2.27E-02	1.242
Cd97	26364	2.22E-02	0.349
Rfwd2	26374	3.32E-02	0.498
Map3k12	26404	1.79E-03	2.622
Mapk9	26420	2.54E-02	1.362
Mmp23	26561	4.56E-07	4.585
Ddx3y	26900	1.39E-02	0.906
Gcat	26912	2.74E-02	-1.106
H2afy	26914	2.14E-02	-0.883
Islr	26968	1.95E-03	2.665
Sh3d19	27059	1.68E-02	3.295
Snord35b	27212	3.02E-03	-2.538
Pdk4	27273	1.25E-02	3.357
Tnfrsf12a	27279	1.83E-02	1.179
Tbl2	27368	4.92E-02	-1.428
Pign	27392	2.42E-02	-1.137
Mrpl15	27395	2.49E-02	0.719
Abca3	27410	5.84E-04	-2.259
1700088E04Rik	27660	3.57E-02	-1.469
Ubfd1	28018	3.27E-02	-0.704
D5Wsu178e	28042	4.52E-02	-1.214
Igfbp7	29817	1.79E-02	1.536
Mapk12	29857	1.96E-02	2.223
Sult4a1	29859	4.90E-02	-1.129
Scmh1	29871	4.26E-02	-1.545
Pcsk1n	30052	1.01E-02	-1.282
Mfi2	30060	2.26E-03	2.580
Fbxl10	30841	7.17E-03	2.391
Slamf6	30925	8.72E-03	-1.367
Ddx25	30959	3.20E-02	0.531
Igbp1b	50540	3.66E-02	-2.507

Fbxl17	50758	1.14E-02	-2.173
Rgs3	50780	3.33E-03	2.067
Nsbp1	50887	9.30E-03	1.655
C1s	50908	1.37E-05	3.713
Rnf24	51902	3.08E-03	-2.455
Coq5	52064	2.98E-03	-1.939
Odf2l	52184	4.53E-02	0.981
Commd2	52245	5.89E-04	2.423
Vps37a	52348	1.31E-02	-1.270
Rcn3	52377	4.96E-02	1.353
Gpr123	52389	3.74E-02	2.475
Echdc2	52430	1.66E-03	3.952
Tax1bp1	52440	3.03E-02	0.625
Ddx56	52513	1.63E-04	-2.345
Mettl2	52686	1.39E-02	-2.017
Gpr172b	52710	1.21E-02	-1.011
Fam3b	52793	1.49E-02	2.629
Lair1	52855	9.73E-03	-1.082
Bysl	53414	2.12E-02	1.304
Snrpa	53607	7.30E-03	2.345
Avpr1a	54140	4.56E-02	2.547
Gucy1b3	54195	2.01E-02	3.984
Arl6ip1	54208	3.27E-02	-0.816
Gp9	54368	2.13E-02	2.889
Nfat5	54446	5.40E-03	2.811
Abhd2	54608	2.75E-02	-1.491
Pdgfc	54635	2.59E-02	-1.872
Gpr97	54672	5.75E-03	-1.839
Hes6	55927	1.40E-02	-1.463
Dclre1a	55947	2.67E-02	1.391
Pdzrn3	55983	2.53E-02	2.092
Fmo2	55990	8.15E-03	1.958
Pgam2	56012	3.36E-02	-1.498
Tusc4	56032	1.75E-02	-1.206
Uqcc	56046	1.68E-02	-1.287
Car5b	56078	1.21E-02	-2.156
Rabggta	56187	1.70E-02	-1.608
Fxyd1	56188	2.10E-02	2.140
Heyl	56198	1.59E-02	2.238
Htra1	56213	5.28E-04	2.721
Actr8	56249	8.02E-03	-1.309
Cpxm1	56264	7.82E-03	1.747
Tmem45a	56277	1.42E-04	3.947
Metap2	56307	2.50E-02	0.699
Nupr1	56312	5.29E-04	1.419
Abcb9	56325	4.74E-03	-0.994
Eif3c	56347	4.38E-02	-0.699
Ivd	56357	2.67E-02	-0.890
Scoc	56367	3.93E-02	-1.617

Apip	56369	4.99E-02	-0.489
B4galt6	56386	3.93E-02	-1.325
Habp4	56541	3.62E-02	-1.245
Ick	56542	3.52E-02	-1.827
Clec7a	56644	4.22E-02	0.530
Psrc1	56742	3.77E-02	-1.773
Pf4	56744	7.09E-03	5.071
Clec1b	56760	3.48E-03	3.259
Med20	56771	2.91E-02	-1.817
Scube2	56788	2.50E-02	2.030
Nelf	56876	7.94E-03	-2.088
Tob2	57259	8.02E-03	-1.095
Fzd2	57265	4.92E-03	3.413
Cxcl14	57266	3.73E-05	3.488
Slurp1	57277	7.76E-04	2.505
Ppbp	57349	4.28E-03	7.414
Gcs1	57377	1.25E-02	-0.594
Xrcc2	57434	7.76E-03	-1.386
S3-12	57435	2.73E-02	1.279
Pglyrp2	57757	9.11E-03	-1.294
Cdon	57810	1.32E-02	-1.669
Tesc	57816	7.76E-03	1.437
Angptl4	57875	1.59E-02	1.052
Fam184b	58227	9.73E-03	-1.839
Nkain4	58237	2.96E-03	-2.523
Efemp2	58859	3.31E-03	3.199
Tssk3	58864	1.17E-04	-2.141
Fam13a	58909	2.31E-03	2.675
Rps6kb2	58988	3.83E-04	-2.860
Wdr8	59002	3.03E-03	-1.328
Pias4	59004	9.64E-03	2.140
Moxd1	59012	2.70E-02	2.484
Slc4a8	59033	1.89E-03	-1.837
Dact1	59036	1.68E-02	2.234
Jph2	59091	1.13E-03	-2.019
Emcn	59308	4.32E-02	1.377
Fads3	60527	4.06E-02	0.834
Trp53inp1	60599	1.25E-02	0.640
Aqp9	64008	3.88E-02	3.484
Smoc1	64075	3.90E-02	2.567
Parvg	64099	2.14E-02	-1.131
Sdf2l1	64136	2.23E-03	-0.951
Ptges	64292	3.31E-02	1.767
Gprc5b	64297	1.66E-02	2.123
Gng13	64337	4.20E-02	-1.853
Svep1	64817	1.03E-03	2.319
Tsc1	64930	3.17E-02	-1.879
B230120H23Rik	65964	4.55E-02	-1.035
Krtcap2	66059	2.75E-02	-0.691

Gng11	66066	2.32E-02	1.327
Chchd6	66098	4.55E-02	-1.091
Tspan13	66109	2.28E-02	-0.868
Mosc1	66112	2.65E-04	4.555
Cml1	66116	9.30E-03	-1.231
Fkbp11	66120	2.50E-02	-1.113
Anapc11	66156	2.39E-06	-2.629
Ccdc72	66167	2.98E-02	-0.503
1110036O03Rik	66180	9.29E-04	3.468
Cd302	66205	2.70E-02	0.784
Sec61b	66212	4.92E-02	-0.544
Klf15	66277	4.62E-02	2.188
Isoc1	66307	4.08E-02	-1.123
Wdr61	66317	2.27E-02	-1.719
Susd3	66329	1.42E-02	-1.917
Ahnak	66395	2.10E-02	0.780
2210417D09Rik	66532	2.52E-02	-1.290
2310050C09Rik	66533	2.71E-02	-2.369
Atpbd4	66632	4.57E-04	3.475
Map1lc3a	66734	2.89E-02	-1.110
Rnf220	66743	3.37E-02	-1.235
Grtp1	66790	2.52E-02	1.208
Bcs1l	66821	4.45E-02	-0.786
0610007N19Rik	66835	1.46E-05	4.547
Dnajc10	66861	2.60E-02	-1.600
1300014I06Rik	66895	4.19E-02	1.020
Rexo1	66932	3.88E-02	-1.694
Zcchc18	66995	1.74E-02	-1.248
1600012F09Rik	67008	3.09E-02	1.180
Oma1	67013	7.58E-03	-1.967
Rpl11	67025	2.64E-02	0.689
Oxct1	67041	2.77E-02	-0.727
Higd2a	67044	2.47E-02	-0.680
Gatm	67092	3.40E-02	-0.847
2510048L02Rik	67119	4.54E-02	2.507
Acbd4	67131	2.47E-02	-0.780
Tomm34	67145	4.24E-02	-1.658
Pdzk1ip1	67182	1.64E-02	1.573
Ndufa13	67184	1.59E-02	-0.464
2810474O19Rik	67246	5.04E-03	0.828
2900005J15Rik	67261	3.13E-02	2.459
Socs4	67296	1.59E-02	-1.839
3110045C21Rik	67303	4.19E-02	-1.614
Fam164a	67306	9.64E-03	2.410
Atp8b3	67331	4.02E-03	3.793
1700084E18Rik	67350	1.39E-02	-1.771
Ppil4	67418	9.04E-05	-2.368
Plxdc2	67448	3.91E-05	3.202
1200011I18Rik	67467	1.95E-02	1.160

Trim62	67525	3.89E-04	4.430
Larp6	67557	3.33E-03	3.215
Rnf41	67588	3.30E-02	1.043
Fibin	67606	7.96E-04	4.315
Anp32b	67628	4.98E-02	0.317
Spc24	67629	8.97E-03	-1.131
Sdhb	67680	4.52E-02	-0.738
3300001P08Rik	67684	2.95E-02	0.812
Aldh3b1	67689	1.67E-02	-0.542
Nsmce1	67711	4.93E-02	-0.569
Nudt13	67725	1.69E-02	2.327
Snap47	67826	1.74E-02	-0.927
Sncaip	67847	1.75E-02	1.677
Echdc3	67856	1.83E-02	1.725
Rgs10	67865	2.79E-02	0.567
Enoph1	67870	3.70E-02	-1.846
Tmem86a	67893	1.96E-02	0.763
Ppap2b	67916	1.89E-03	1.942
Tmem59l	67937	1.95E-03	-1.571
Atp5g2	67942	2.14E-02	-0.395
Atad1	67979	1.46E-02	1.345
Cox19	68033	1.47E-02	-0.882
Arpc4	68089	4.76E-02	-0.856
Yif1a	68090	4.93E-02	-1.601
Mum1	68114	3.93E-02	1.253
Cgnl1	68178	9.30E-03	-5.429
Chchd8	68185	7.43E-04	-1.464
Gtsf1l	68236	3.02E-02	-1.779
Toe1	68276	1.61E-03	2.642
Crip2	68337	3.79E-02	0.794
G6pc3	68401	4.70E-02	-0.711
Dhrs7c	68460	1.98E-02	-1.698
1110018H23Rik	68509	1.45E-03	-2.128
Gpr155	68526	9.73E-03	1.288
Caly	68566	2.71E-04	-1.672
Abhd14a	68644	5.81E-03	-2.264
Fndc1	68655	3.79E-08	5.438
Scgb3a1	68662	2.31E-04	3.169
Slc44a2	68682	1.68E-02	-1.170
Lce1b	68720	1.64E-02	2.273
Dus1l	68730	1.19E-02	2.390
Tmem219	68742	2.90E-02	1.228
Atp6v1c2	68775	2.16E-04	4.205
Flnc	68794	4.43E-02	2.324
Ubr3	68795	7.58E-03	2.434
Ppil1	68816	3.29E-02	-0.934
Tmcc2	68875	9.26E-03	2.644
Mitd1	69028	1.21E-02	2.392
4930579G22Rik	69034	2.37E-02	-1.820

Tmem121	69195	1.91E-02	-0.917
Sat2	69215	1.33E-02	2.843
2610034M16Rik	69239	1.72E-02	2.367
1700008P20Rik	69301	2.41E-05	-2.858
Ubl7	69459	3.27E-02	2.204
Antxr1	69538	1.27E-05	3.135
Vkorc1l1	69568	1.70E-02	2.362
Mfsd3	69572	3.25E-02	-0.994
Gpx8	69590	2.73E-06	4.056
2310043J07Rik	69665	4.28E-02	-1.775
Iqcg	69707	1.29E-02	1.752
Ttl	69737	1.42E-02	-2.138
1500009L16Rik	69784	1.40E-02	2.171
Krtcap3	69815	3.76E-02	-0.845
2010001M09Rik	69816	3.94E-03	-1.404
Glod5	69824	7.73E-03	2.754
Ttc39b	69863	3.20E-03	1.709
1810065E05Rik	69864	5.82E-03	-2.043
Mfsd11	69900	4.21E-02	2.035
Dnahc17	69926	3.46E-03	-1.933
Scrn1	69938	3.86E-03	-2.706
2810011L19Rik	69952	1.12E-03	-2.291
Cdc16	69957	8.21E-03	-2.001
1700026L06Rik	69987	6.30E-04	-1.621
2010309G21Rik	70018	1.16E-03	0.953
Acot7	70025	2.37E-02	-0.741
Trnt1	70047	4.43E-03	-2.860
Ccdc89	70054	3.81E-03	-1.934
Metrn	70083	2.30E-02	-0.757
Cyp4f16	70101	2.29E-02	3.773
Ufsp1	70240	7.17E-03	1.168
Derl3	70377	2.78E-02	-1.556
Tbce	70430	4.20E-02	2.025
Wars2	70560	1.96E-02	-1.633
5730469M10Rik	70564	2.08E-02	0.620
Tmem144	70652	1.07E-02	2.211
Gpr125	70693	2.43E-02	2.151
Kynu	70789	1.37E-02	-1.894
D19Ertd652e	70806	6.48E-06	3.528
Hmgxb4	70823	3.48E-02	1.254
Mmrn1	70945	1.94E-02	2.439
4931408A02Rik	70967	4.78E-02	1.700
Tssk4	71099	3.93E-02	1.692
4933407H18Rik	71101	1.22E-02	-2.058
4933407L21Rik	71141	2.32E-05	-2.857
1700112M01Rik	71184	1.43E-03	-2.355
4933440M02Rik	71208	4.32E-02	-1.567
4933429H19Rik	71280	4.81E-02	1.525
Arhgap26	71302	2.25E-02	-2.050

Treml1	71326	5.35E-03	4.628
Flrt3	71436	2.22E-02	1.462
Tmem80	71448	1.03E-02	-1.803
9030409G11Rik	71529	6.76E-03	2.520
9030425E11Rik	71566	4.86E-05	2.393
Senp8	71599	2.19E-02	-1.734
Rarres2	71660	2.33E-05	3.216
Esm1	71690	3.08E-07	3.774
Syde1	71709	4.20E-02	2.028
Ranbp3	71810	3.16E-02	-1.259
Pdia6	71853	3.91E-02	-1.171
Serpinb12	71869	2.02E-02	-1.913
Noxo1	71893	4.10E-02	1.474
Lypd6b	71897	1.42E-02	-1.244
2310028H24Rik	71901	7.09E-03	-1.313
Scand3	71970	9.89E-03	1.567
Rbpms2	71973	1.53E-02	1.737
Pgm2	72157	4.84E-02	-0.670
Dbndd1	72185	2.61E-03	-2.729
Cda	72269	3.61E-02	1.825
Nkg7	72310	1.05E-04	-0.988
Palld	72333	6.49E-05	1.541
Fam164c	72350	1.87E-02	2.075
Rnf219	72486	1.20E-02	-1.903
Spats2	72572	1.81E-03	2.857
2810006K23Rik	72650	1.78E-02	-1.515
Serp2	72661	4.19E-03	-1.218
Zfp74	72723	1.31E-02	2.360
B3gat3	72727	2.57E-03	-1.646
2810410P21Rik	72731	2.38E-02	3.583
Hepacam	72927	1.40E-02	-2.260
2900019G14Rik	72932	7.17E-03	-2.078
Insig2	72999	1.56E-03	1.459
Camk2n2	73047	2.82E-02	-0.941
Slc22a23	73102	1.01E-03	1.179
Prssl1	73106	1.68E-02	-0.777
Tm7sf2	73166	2.39E-02	-1.153
Pcdh18	73173	1.42E-04	2.904
Pear1	73182	1.29E-04	-2.530
3110043A19Rik	73216	3.77E-02	3.052
Bmper	73230	6.41E-03	4.138
Ddit4l	73284	1.39E-02	2.298
Rhobtb3	73296	3.04E-02	2.285
1700054F22Rik	73398	1.99E-02	3.714
1700054N08Rik	73420	3.36E-02	1.616
Rnf38	73469	3.60E-02	-1.795
Trp53tg5	73603	2.35E-02	-3.254
2410089E03Rik	73692	2.28E-02	-2.849
Fam125a	73711	1.93E-02	-0.702

Whrn	73750	4.20E-02	-1.623
1110012D08Rik	73827	4.01E-02	2.338
Otud4	73945	2.32E-02	-1.050
Slc25a27	74011	4.28E-02	2.749
Msl1	74026	1.25E-02	-1.979
4931406B18Rik	74054	2.90E-04	-2.582
4933407I05Rik	74072	4.56E-02	2.910
Paqr5	74090	8.02E-03	-1.418
Cep55	74107	2.03E-02	-1.893
Errfi1	74155	2.48E-03	1.745
Prei4	74182	3.48E-03	1.358
Phactr3	74189	3.09E-02	-1.126
1200009I06Rik	74190	2.50E-02	1.628
Vit	74199	5.16E-04	2.133
Spatc1	74281	4.86E-02	2.166
Ranbp10	74334	4.50E-02	0.714
Xrcc3	74335	4.72E-02	-1.081
Zfp84	74352	3.35E-02	1.972
4932417H02Rik	74370	2.89E-02	2.211
Snx29	74478	2.81E-03	-2.182
Samd4	74480	3.49E-02	-1.642
Lrrc17	74511	2.25E-02	1.957
Morc2a	74522	1.07E-02	-1.833
4833422F24Rik	74614	2.25E-02	-1.835
4930507D05Rik	74706	9.51E-05	-2.315
Trim14	74735	2.41E-02	-1.741
Atp13a2	74772	3.29E-02	0.647
Wipi2	74781	2.79E-02	-1.225
4930506C21Rik	75060	9.00E-03	-2.139
4930517O19Rik	75075	8.59E-05	2.901
Uhrf1bp1l	75089	3.91E-02	-1.664
4930520O04Rik	75116	9.01E-04	-2.309
Tmem180	75146	1.26E-02	-0.867
Sv2c	75209	9.60E-03	-1.796
Dusp18	75219	1.64E-02	1.954
Fgfr1op	75296	1.45E-02	-1.922
3100002J23Rik	75429	9.89E-03	-2.521
Phpt1	75454	1.74E-02	-0.531
1700011E24Rik	75467	2.87E-03	-2.242
Zbtb4	75580	4.13E-02	1.547
Rai14	75646	2.15E-03	3.664
Fam148b	75697	4.89E-03	-1.996
Tmem14a	75712	6.03E-03	-1.248
Them4	75778	4.50E-02	-1.661
Clec4g	75863	6.15E-04	-2.058
Slain2	75991	4.20E-02	2.058
6230409E13Rik	76132	3.59E-02	-2.566
Slc35d3	76157	1.98E-03	3.502
Med13l	76199	2.22E-02	-2.039

Jakmip2	76217	5.44E-03	-2.062
Atp6v0e2	76252	7.62E-08	-4.159
Gpt	76282	2.08E-02	-1.249
1110021L09Rik	76306	3.65E-02	1.422
1700012L04Rik	76383	3.82E-02	1.249
1700019E19Rik	76411	8.40E-03	1.193
Prss23	76453	2.65E-04	2.945
Ly6k	76486	1.38E-02	-2.277
Il34	76527	4.55E-02	1.212
1700125G22Rik	76657	1.20E-02	4.245
Clasp1	76707	3.30E-02	-1.704
Snx27	76742	2.69E-02	-1.141
Mospd2	76763	2.13E-02	1.446
2010005H15Rik	76770	2.93E-02	-1.171
Klhdc10	76788	2.33E-02	-0.953
Calcoco2	76815	3.01E-02	2.067
Ccdc116	76872	1.69E-02	-1.640
Rab36	76877	2.49E-03	-2.095
Scfd1	76983	9.93E-03	-1.915
5730590G19Rik	77011	1.07E-02	-1.497
Fam162b	77296	1.29E-04	-2.528
Kidins220	77480	2.87E-02	-2.000
4931406H21Rik	77592	3.28E-04	-2.535
Usp45	77593	2.34E-02	1.137
Nup210l	77595	2.05E-02	2.051
H2afv	77605	3.51E-02	-0.402
Elp4	77766	1.87E-02	-1.705
A930004D18Rik	77940	8.81E-04	-2.847
Prr15	78004	1.19E-02	1.362
Klhdc8b	78267	2.07E-02	1.498
Mtap7d2	78283	3.64E-02	-1.891
2700046A07Rik	78449	1.24E-02	2.961
Skap1	78473	1.32E-02	-1.391
Tmsl8	78478	3.72E-02	-1.761
Htra3	78558	3.85E-04	3.039
Lsm6	78651	2.63E-02	-1.216
Usp54	78787	2.47E-02	2.565
9630013K17Rik	78883	3.18E-02	3.170
Igsf3	78908	4.52E-04	1.377
Nadsyn1	78914	4.20E-02	-0.850
4930581F22Rik	78934	2.43E-02	2.371
Rab27b	80718	3.12E-02	2.985
Mynn	80732	4.56E-04	-2.512
Ghdc	80860	3.57E-02	-1.526
Cxcr6	80901	2.94E-02	-1.530
Cyp4x1	81906	1.19E-02	2.392
Glis2	83396	3.36E-03	2.957
Gimap3	83408	2.79E-02	-2.203
Chrdl1	83453	3.45E-05	3.787

Fthl17	83457	3.68E-02	-1.290
Tdrd1	83561	3.55E-06	-2.498
Bicc1	83675	1.99E-05	3.993
Pde4dip	83679	9.93E-03	-2.011
Smarcd2	83796	4.99E-02	-0.916
Rnf123	84585	4.36E-03	1.812
Pla1a	85031	8.12E-03	1.950
Pard3	93742	4.63E-02	1.828
Pcdhb4	93875	1.31E-02	-1.792
Pcdhb22	93893	5.61E-03	2.776
Nkd1	93960	7.38E-05	3.575
Trim9	94090	3.48E-02	-1.628
Nans	94181	4.74E-02	-0.666
Ophn1	94190	4.52E-02	1.682
S1pr5	94226	2.29E-02	-1.978
Loxl2	94352	2.54E-03	2.130
Nhedc2	97086	2.05E-03	-2.094
D2hgdh	98314	1.37E-03	-2.391
Zfp451	98403	4.33E-02	1.359
Obsl1	98733	3.16E-04	5.130
Myl9	98932	2.00E-04	3.240
Anapc2	99152	2.37E-02	-1.921
Garnl3	99326	4.33E-04	-2.153
Olfml3	99543	2.94E-02	2.381
Lphn2	99633	3.19E-02	0.979
Osbpl9	100273	4.78E-02	-1.313
Bsdc1	100383	1.04E-02	2.033
Trrap	100683	3.21E-02	2.049
Tada3l	101206	5.03E-05	-3.172
Adamts9	101401	2.13E-03	2.691
Wtip	101543	1.44E-03	2.409
Nlrp6	101613	4.98E-02	3.243
Psip1	101739	4.49E-02	0.658
Plekhg4	102075	3.65E-02	-1.082
2310065K24Rik	102122	7.85E-03	-2.472
Taf5l	102162	1.93E-02	2.060
Pls1	102502	3.86E-03	4.007
Alg9	102580	4.06E-03	-2.024
Armcx4	102910	4.98E-02	1.924
Upb1	103149	2.07E-02	-0.858
BC030307	103220	7.77E-03	-2.649
Fam26e	103511	1.50E-02	2.824
Mbtd1	103537	2.14E-02	1.837
Qsox1	104009	1.01E-02	-1.786
Adcy4	104110	3.49E-02	1.778
Acly	104112	2.99E-02	-1.023
Cnot6	104625	4.20E-02	0.602
1110002B05Rik	104725	2.83E-02	-0.686
Fam110c	104943	3.21E-02	2.133

Epdr1	105298	1.86E-02	1.194
Ypel1	106369	2.29E-02	-1.954
Gpr137	107173	4.23E-02	-1.538
Kazald1	107250	6.97E-04	2.149
Brms1	107392	1.08E-02	-1.325
Mylk	107589	5.22E-03	2.453
Rapgef1	107746	3.65E-02	-1.777
Scamp1	107767	2.54E-02	-1.255
Mgat5	107895	1.38E-03	-2.395
Celsr3	107934	2.23E-02	1.945
Bfsp2	107993	8.02E-06	-1.634
Brunol4	108013	2.12E-03	-2.904
Slc14a1	108052	2.06E-03	1.482
Adamts7	108153	7.76E-03	1.105
Chn1	108699	7.76E-03	-2.057
Galntl1	108760	8.12E-03	-1.893
Ston2	108800	2.59E-03	2.523
Rcc2	108911	3.75E-02	-0.553
Cdca2	108912	2.36E-02	1.531
E2f8	108961	3.77E-02	-0.788
Lrrc39	109245	2.57E-03	2.892
C1qtnf7	109323	2.03E-02	4.269
Cald1	109624	1.84E-02	3.015
Acy1	109652	4.99E-02	-1.658
Hyal3	109685	6.76E-03	-1.518
Maob	109731	3.70E-02	3.321
Mzf1	109889	4.78E-02	-1.124
Mpi	110119	1.91E-02	-1.382
Fdps	110196	3.22E-02	-1.302
Akr7a5	110198	2.25E-02	-1.407
Hba-a2	110257	3.58E-03	0.869
Bcr	110279	1.13E-03	-2.583
Pigh	110417	1.55E-02	-2.125
Hivep1	110521	1.52E-02	1.495
Adarb1	110532	4.77E-02	1.855
Amhr2	110542	1.09E-02	3.211
Rps6ka3	110651	4.79E-02	1.043
Pcca	110821	4.28E-02	-1.266
Erc1	111173	4.66E-02	-1.090
Igh	111507	4.65E-07	-1.927
Acaa1a	113868	8.89E-03	-1.413
Elac1	114615	6.06E-03	2.237
Dscaml1	114873	3.92E-04	3.899
Plcz1	114875	3.49E-03	-1.893
Cygb	114886	3.39E-02	1.795
Prelp	116847	1.38E-02	2.259
Cspg4	121021	3.34E-03	-2.096
Plxnb2	140570	3.15E-02	2.190
Myh7	140781	8.89E-04	-2.115

Caln1	140904	9.36E-05	-3.485
Elovl6	170439	2.74E-02	-0.981
Grin3b	170483	2.73E-02	-1.632
Pfkfb3	170768	3.79E-02	0.998
Cd209d	170779	4.19E-03	-1.843
Acot8	170789	3.10E-02	-0.613
AY036118	170798	8.89E-03	1.476
Eraf	170812	1.07E-03	0.834
Ms4a3	170813	7.76E-03	-0.682
Glmn	170823	4.77E-02	1.770
Tram2	170829	1.42E-02	-1.631
Inpp5j	170835	5.07E-03	-1.965
Il17rc	171095	7.43E-04	2.249
Edaradd	171211	1.04E-02	-2.236
V1rh7	171250	4.28E-02	1.892
Gpr37l1	171469	4.59E-05	-3.623
Bspry	192120	2.01E-02	1.920
Stab1	192187	1.02E-02	1.433
Zfp286	192651	3.29E-03	-2.221
Pcdhg@	192682	1.54E-02	-1.669
AB182283	192950	1.27E-03	2.531
Zbtb12	193736	1.96E-02	-1.064
Hspa1a	193740	4.25E-02	1.843
Rimkla	194237	4.37E-05	-2.666
Sec23ip	207352	2.25E-03	-2.181
Tbc1d16	207592	9.71E-03	-2.205
Thsd4	207596	3.76E-03	3.357
Phldb2	208177	3.65E-02	3.167
Etl4	208618	1.88E-02	2.128
Fam20a	208659	6.83E-03	2.317
Dis3l2	208718	2.25E-02	-2.005
Sned1	208777	1.57E-02	1.617
Daam1	208846	2.37E-02	1.668
Npb	208990	1.95E-02	-1.774
Gan	209239	1.40E-02	2.091
Enpp3	209558	4.69E-02	2.363
Lcorl	209707	4.99E-02	-1.404
Tmc7	209760	9.73E-03	0.983
Pgbd5	209966	8.37E-04	-2.533
Tmem194	210035	3.29E-02	-1.118
Adcy2	210044	9.30E-03	2.643
Mtrf1	211253	5.69E-03	1.379
EG211331	211331	4.23E-06	-2.193
Tfdp2	211586	8.11E-03	1.151
Plac9	211623	6.55E-06	2.787
Mgst2	211666	2.54E-02	-0.561
Arfgef1	211673	3.40E-02	-1.247
Vstm2a	211739	0.00E+00	-4.085
Hk3	212032	4.26E-02	-0.636

2610015P09Rik	212153	3.02E-02	-0.993
Ubxn10	212190	2.61E-02	3.324
Paox	212503	3.74E-02	-0.652
Sprn	212518	5.04E-03	-2.083
Scfd2	212986	4.85E-02	-0.854
Fstl5	213262	7.17E-03	-1.831
Fbxl21	213311	4.97E-02	2.418
Rassf4	213391	3.83E-02	0.883
Gpr174	213439	2.94E-02	3.069
Dstyk	213452	8.21E-03	2.108
Nudt18	213484	5.82E-03	-2.429
Prepl	213760	3.38E-02	-1.652
Tmem51	214359	2.47E-02	0.715
Cdk5rap2	214444	2.87E-02	-1.283
Aldh5a1	214579	2.03E-02	-1.170
Spg11	214585	2.22E-02	-1.587
Mobkl2b	214944	2.47E-02	-1.510
Sema6d	214968	6.28E-03	2.446
Bud13	215051	3.70E-02	-1.153
Fcgbp	215384	4.17E-02	2.018
Ncaph	215387	7.52E-03	-1.618
Rassf2	215653	3.96E-02	0.892
Nhsl1	215819	4.96E-02	1.087
Hkdc1	216019	6.23E-05	-3.416
Pdxk	216134	2.26E-02	-1.893
Wdr18	216156	2.92E-02	-1.675
ORF61	216157	2.13E-02	-1.146
Socs2	216233	1.59E-05	3.588
Efemp1	216616	3.57E-04	3.958
Adamts2	216725	1.47E-05	3.180
Centb1	216859	2.94E-02	-0.932
Adap2	216991	4.98E-02	1.545
Unc45b	217012	1.26E-02	1.585
Heatr6	217026	2.25E-02	-0.904
OTTMUSG00000002043	217122	3.91E-02	-1.758
Samd14	217125	1.97E-03	1.362
Scrn2	217140	2.21E-06	-2.785
Abca9	217262	2.85E-02	1.245
Cd300lb	217304	6.60E-03	2.561
Myo15b	217328	2.49E-02	-1.650
Snx13	217463	2.26E-02	1.984
Rapgef5	217944	1.95E-02	1.059
Heatr1	217995	1.12E-02	-1.840
Pou6f2	218030	3.56E-02	-1.476
Amph	218038	2.00E-05	-2.983
Mylip	218203	5.05E-03	0.888
Serinc5	218442	1.23E-02	-3.094
Paip1	218693	3.89E-02	-1.653
Wapal	218914	7.82E-03	0.899

Fermt2	218952	1.14E-02	1.608
9130227C08Rik	219094	4.66E-02	-1.585
Scara3	219151	1.22E-02	2.856
Farp1	223254	4.25E-02	0.848
E430025E21Rik	223593	4.01E-02	-1.652
Nrbp2	223649	3.44E-02	1.755
Dbx2	223843	1.14E-05	-2.526
Uhrf1bp1	224648	1.69E-03	-2.159
Bat3	224727	1.96E-02	-1.956
Gpr116	224792	2.37E-02	1.976
Aars2	224805	2.04E-02	-1.252
AI661453	224833	1.76E-02	-1.205
Gm546	224908	2.50E-02	2.255
Ttc7	225049	1.10E-02	-1.835
Mib1	225164	3.60E-02	-0.988
Wdr36	225348	2.45E-02	-1.783
Suv420h1	225888	3.82E-02	1.306
Dak	225913	5.07E-03	-1.703
Pgm5	226041	1.06E-02	2.252
Fam160b1	226252	4.17E-02	-0.752
Smg7	226517	3.17E-02	-1.322
Gm106	226866	3.81E-02	-3.324
Plekhb2	226971	3.12E-02	-1.911
Eif5b	226982	2.73E-02	0.765
Ccnyl1	227210	1.20E-02	1.713
Rpp38	227522	6.98E-04	2.228
5430407P10Rik	227545	1.35E-02	3.598
Slc25a25	227731	2.41E-02	0.824
Rabgap1	227800	6.67E-03	2.699
Ccdc148	227933	8.02E-06	-2.451
Agps	228061	3.77E-02	-0.923
Slc35c1	228368	3.79E-02	-0.542
Prrg4	228413	2.37E-02	-1.658
Vps18	228545	1.12E-02	-1.562
Mall	228576	7.64E-04	2.925
Prkcbp1	228880	1.96E-02	-0.863
Isg20l2	229504	3.40E-02	1.037
Syt11	229521	8.76E-07	-2.780
Msto1	229524	2.16E-02	-0.810
Vangl1	229658	2.21E-02	1.189
Bcl2l15	229672	1.59E-02	-0.989
Rsbn1	229675	2.84E-02	-1.759
D3Bwg0562e	229791	2.24E-04	-2.682
Ccbl2	229905	1.38E-02	-1.486
E130306D19Rik	230098	3.20E-02	-1.137
AI314180	230249	2.02E-02	-1.789
Leprot	230514	1.25E-02	0.986
Cyb5rl	230582	9.61E-03	-1.395
Jmjd2a	230674	8.11E-03	2.689

C230096C10Rik	230866	4.62E-02	-0.590
Igsf21	230868	4.75E-03	-2.072
Phf13	230936	2.02E-03	-2.886
9430015G10Rik	230996	4.10E-02	3.051
Dok7	231134	3.60E-02	1.420
Slc10a4	231290	3.32E-02	-1.780
Uba6	231380	3.53E-02	3.263
A830010M20Rik	231570	2.03E-02	-2.725
C330023M02Rik	231713	2.05E-02	1.883
Rimbp2	231760	3.05E-03	-2.071
Amz1	231842	2.89E-02	-0.952
Fam176a	232146	2.61E-02	1.660
Frmd4b	232288	4.99E-02	1.576
Glt8d4	232313	8.71E-03	2.498
Ankrd26	232339	1.75E-03	1.674
Crebl2	232430	1.08E-02	-1.369
Rerg	232441	7.80E-03	2.776
Ccdc106	232821	3.12E-02	-1.731
Samd4b	233033	1.36E-03	1.768
Smg1	233789	3.68E-02	1.941
Thumpd1	233802	4.38E-02	-1.880
D430042O09Rik	233865	8.40E-03	2.375
Asphd1	233879	2.14E-04	-2.486
Ppfia1	233977	2.74E-02	1.478
Arglu1	234023	1.34E-03	0.844
Pcid2	234069	4.92E-03	-2.213
Yjefn3	234365	1.06E-02	-1.383
Sfrs14	234373	7.53E-03	-2.297
Ndrg4	234593	1.43E-02	-2.445
BC015286	234669	3.59E-05	4.509
Ftsjd1	234728	4.66E-02	-1.635
Vac14	234729	2.92E-02	1.663
Ddx19b	234733	4.08E-02	-1.331
Slc38a8	234788	1.42E-02	-3.750
Fam38a	234839	3.69E-02	0.503
Nup133	234865	2.69E-02	0.575
BC050092	235048	4.10E-02	-1.181
Rnf214	235315	2.96E-03	-2.023
Sik2	235344	2.10E-02	1.579
Cd109	235505	8.91E-06	3.220
Atp2c1	235574	3.14E-02	-1.704
Dusp7	235584	4.66E-02	-0.753
Zfp445	235682	3.53E-02	-1.884
Eif2c1	236511	1.96E-02	2.848
LOC236598	236598	2.63E-02	1.240
Ddx26b	236790	4.53E-02	0.471
Slc9a6	236794	4.98E-02	0.855
Pctk2	237459	2.48E-02	-1.917
4932414J04Rik	237694	3.80E-02	-1.673

Smcr8	237782	1.60E-02	-1.688
Ccdc55	237859	5.50E-03	0.941
Btbd7	238386	1.66E-03	3.739
Serpina3f	238393	1.95E-02	1.456
Mtr	238505	8.47E-03	-2.233
C330011K17Rik	238692	3.21E-02	1.569
Pde4d	238871	6.59E-03	-1.928
Ogdhl	239017	2.28E-02	3.680
Setdb2	239122	2.93E-02	-1.689
Slc2a13	239606	4.10E-02	2.686
Pdzrn4	239618	1.13E-03	3.546
AI836003	239650	2.08E-02	2.730
Mkl2	239719	1.21E-02	-2.253
Ccdc14	239839	3.22E-02	-1.741
A630033E08Rik	240041	5.42E-03	2.077
Mmp25	240047	2.52E-02	-0.905
Thada	240174	1.80E-04	3.261
9430020K01Rik	240185	8.49E-04	1.768
Slc4a9	240215	6.75E-03	1.782
Ythdc2	240255	4.54E-03	2.419
Sulf1	240725	1.92E-03	3.978
Adamts4	240913	4.56E-02	2.317
EG240921	240921	3.40E-02	-1.239
Zfp385b	241494	2.14E-02	2.164
Fermt1	241639	3.53E-08	-3.389
6330439K17Rik	241688	7.88E-05	-2.441
Igsf10	242050	1.70E-04	2.891
Pde5a	242202	2.14E-02	3.446
Bank1	242248	3.65E-02	-0.986
Lingo2	242384	1.58E-06	-2.848
D630039A03Rik	242484	2.43E-02	-1.631
Atg4c	242557	9.69E-03	-1.892
Wdr78	242584	1.53E-03	-1.875
Tmem130	243339	2.31E-03	-1.697
Lrrc61	243371	1.96E-02	1.832
Clec1a	243653	2.41E-02	2.013
Ppp1r9a	243725	4.26E-02	-1.084
Zfp536	243937	3.13E-02	-1.606
Dlgap2	244310	4.50E-03	-2.013
Rpgrip1l	244585	2.26E-02	-1.904
Gm505	244666	3.88E-02	-1.547
Disc1	244667	8.12E-03	1.564
2210010B09Rik	244721	7.17E-03	1.471
Olfm2	244723	2.33E-02	-1.712
Zc3h12c	244871	2.96E-02	1.032
9930111J21Rik	245240	3.39E-02	1.793
A630018P17Rik	245695	4.66E-02	0.837
Ift52	245866	3.53E-02	2.024
Vasn	246154	5.42E-03	3.366

Klhl8	246293	1.68E-02	-1.883
Lgi2	246316	2.51E-07	-2.954
Oas3	246727	5.18E-03	-1.958
Olfr860	258521	2.22E-02	1.950
Olfr700	258593	1.69E-02	-3.281
Olfr344	258621	2.67E-02	2.207
Nphp4	260305	4.10E-05	-3.205
Spata2	263876	3.52E-02	-1.340
E130112L23Rik	268739	1.21E-03	1.604
Pkmyt1	268930	2.60E-02	-0.847
Wdr86	269633	1.18E-03	3.204
Tspan12	269831	8.80E-03	2.280
2610024B07Rik	269987	4.64E-02	1.448
Vat1l	270097	2.35E-02	-1.815
Rpl13	270106	1.40E-02	-1.754
Vstm2l	277432	1.45E-04	-2.743
Gpr107	277463	4.24E-02	1.742
C2cd3	277939	4.45E-02	-1.060
Pcdh19	279653	4.13E-02	2.086
Snhg11	319317	6.76E-03	-2.488
B930096F20Rik	319332	8.40E-03	2.160
Ppm1h	319468	1.68E-02	-2.143
Itga11	319480	3.30E-04	3.489
Nrcam	319504	5.89E-03	-1.858
7530428D23Rik	319506	3.91E-05	3.956
Dusp4	319520	1.04E-02	1.295
D330013E07Rik	319525	5.49E-04	2.595
C230012O17Rik	319564	4.74E-02	-1.543
Syne2	319565	1.81E-02	-1.714
6430573F11Rik	319582	2.27E-02	-1.710
Rab9b	319642	4.34E-02	-1.648
Usp37	319651	4.71E-02	-0.984
E330037M01Rik	319775	5.49E-04	2.835
Cobll1	319876	1.75E-02	-1.366
Zdhhc17	320150	7.89E-03	1.891
Lrrc58	320184	4.52E-02	-0.567
Ttll5	320244	5.28E-04	2.441
6720469N11Rik	320339	2.85E-02	2.290
Cenpt	320394	2.90E-02	-2.307
Rinl	320435	3.65E-02	-0.832
Heatr5a	320487	3.02E-02	-1.548
Dennd5b	320560	1.66E-03	2.732
Iffo1	320678	4.65E-03	-1.850
C530043K16Rik	320715	3.01E-02	3.040
Pkn1	320795	2.96E-02	-1.215
Amigo3	320844	2.84E-02	1.187
6430562O15Rik	320893	9.73E-03	2.716
Wscd2	320916	6.48E-03	-1.934
Ccbe1	320924	3.02E-02	1.708

Serac1	321007	4.72E-02	-1.554
6720458F09Rik	328162	4.53E-02	-0.891
Parp4	328417	2.74E-02	-1.057
Prss34	328780	6.23E-04	-0.870
Atg2a	329015	2.34E-02	-1.896
9130024F11Rik	329160	2.68E-02	1.848
5230400M03Rik	329406	6.41E-03	2.641
Col8a2	329941	7.50E-03	2.134
BC038925	330216	2.66E-03	-1.754
9530028C05	330256	6.59E-03	0.627
Fbxo41	330369	3.64E-02	-2.083
D630042F21Rik	330428	1.95E-02	1.178
Mtmr15	330554	2.47E-02	2.066
EG333452	333452	2.13E-02	1.734
Nlrp12	378425	3.02E-03	-2.400
Intu	380614	3.61E-02	1.023
Ccnjl	380694	2.14E-02	1.479
AI324046	380795	4.23E-06	-4.399
Unc93a	381058	4.46E-02	-1.088
Fam82a1	381110	3.66E-02	2.304
Nsl1	381318	1.99E-02	-1.462
Trim55	381485	2.92E-02	0.748
Ssbp1	381760	4.76E-05	-2.848
Tatdn2	381801	4.31E-02	-0.922
B230311B06Rik	381914	1.58E-06	-2.918
Taok2	381921	3.19E-02	1.133
Fndc5	384061	2.23E-02	-1.711
Gm1502	385120	1.97E-03	-2.372
Gm1524	385253	5.53E-04	-2.818
Thoc6	386612	4.22E-02	-0.871
Map4k5	399510	4.13E-02	0.887
Flrt2	399558	5.14E-05	3.398
Plcxd1	403178	3.61E-04	-2.348
Iqgap3	404710	3.88E-02	-0.879
Bex4	406217	2.72E-03	1.296
Krt83	406219	2.29E-02	-1.666
BC066028	407812	4.47E-02	-1.527
Akr1c19	432720	8.37E-04	4.298
Ly6g6f	433099	1.96E-02	4.498
LOC435145	435145	2.90E-02	-1.799
LOC435333	435333	3.70E-06	-2.191
Dnm3os	474332	2.61E-03	2.818
Npcd	504193	1.12E-02	3.209
Kalrn	545156	1.92E-02	1.696
Tceal3	594844	3.70E-03	-1.511
Ndufs5	595136	3.68E-03	-0.681
621968	621968	2.89E-02	-1.661
4631416L12Rik	622434	2.38E-02	-1.820
Igk-V21-4	626347	2.59E-06	-3.431

LOC631981	631981	9.64E-03	2.593
LOC634100	634100	3.33E-03	2.281
LOC638038	638038	1.84E-02	-1.675
LOC639988	639988	1.43E-04	-2.064
LOC640979	640979	1.32E-02	-1.802
Tect1	654470	2.22E-02	-1.046
D630032N06Rik	654810	2.12E-02	-2.095
OTTMUSG00000016543	665001	3.39E-02	-1.791
LOC677447	677447	4.49E-02	0.728
LOC677484	677484	5.07E-05	-1.745
4732444A12Rik	100015211	3.09E-11	-3.593
OTTMUSG00000003947	100038570	6.51E-03	2.311
F420014N23Rik	100038591	4.19E-03	-1.945
100041143	100041143	4.43E-02	3.547
ENSMUSG00000071724	100041269	1.18E-02	-1.384
100043189	100043189	3.98E-02	1.807
Srcap	100043597	1.91E-02	0.956
LOC100047053	100047053	4.19E-03	-1.945
LOC100049077	100049077	4.66E-02	-1.378
ENSMUSG00000071525	100049155	1.97E-03	2.739
