# Supplementary material for: Transcriptional landscape of bone marrow-derived very small embryonic-like stem cells during hypoxia
Source: Respir Res. 2011 May 10;12(1):63. doi: 10.1186/1465-9921-12-63 (PMC3098802; doi:10.1186/1465-9921-12-63)

Supplementary Figure. Gene product interaction network of differentially expressed genes in VSELs after *in vivo* exposure to hypoxia. Panel (A) highlights up and downregulated genes (red and green respectively). This network has 424 genes (nodes) and 604 connections (edges). Note that each connection represents a known direct interaction between two gene products. Panel (B) demonstrates that the topology of this network is scale-free because it follows a power law distribution. N_k, degree distribution; k, nodal connectivity.

A)

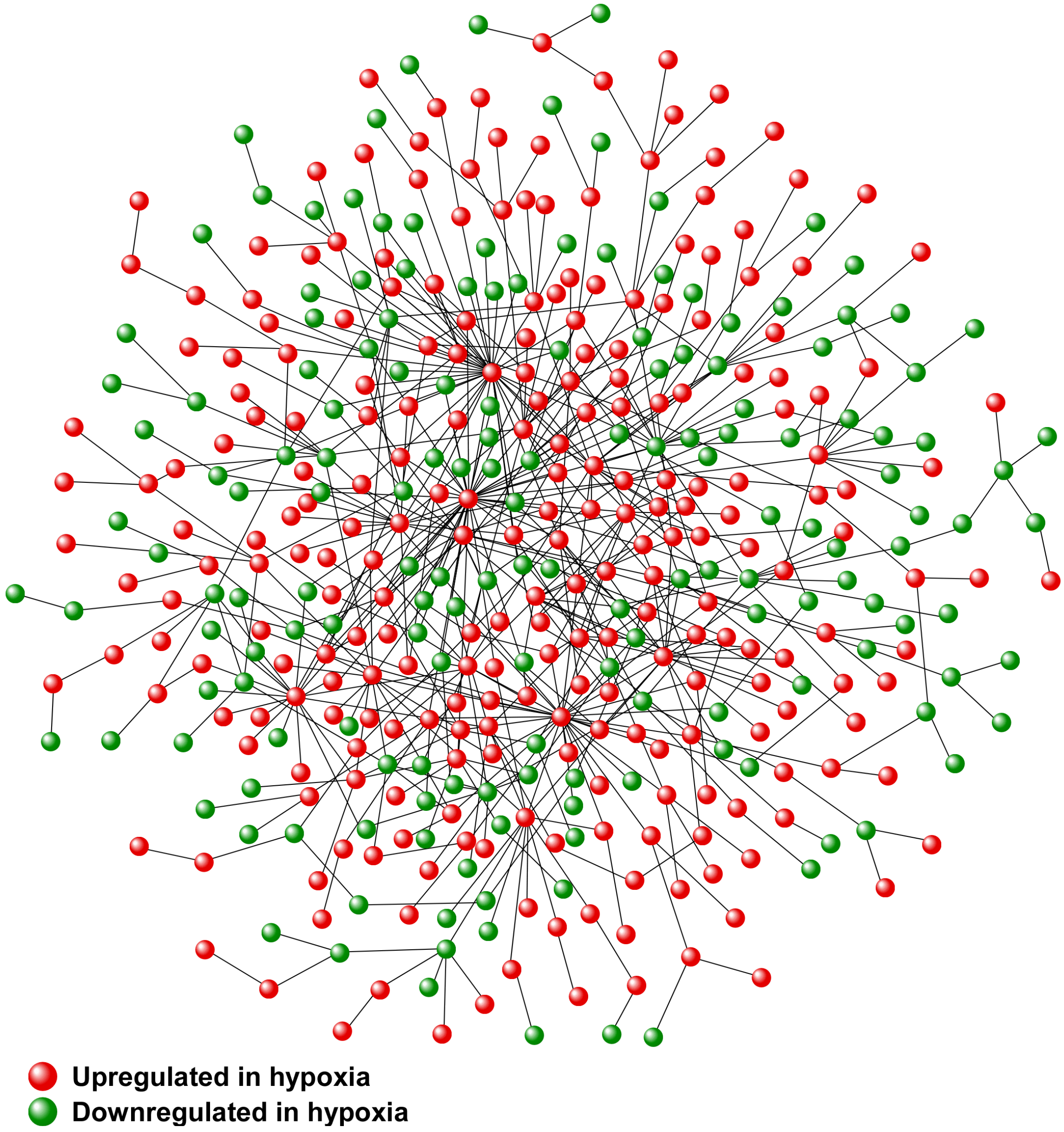

B)

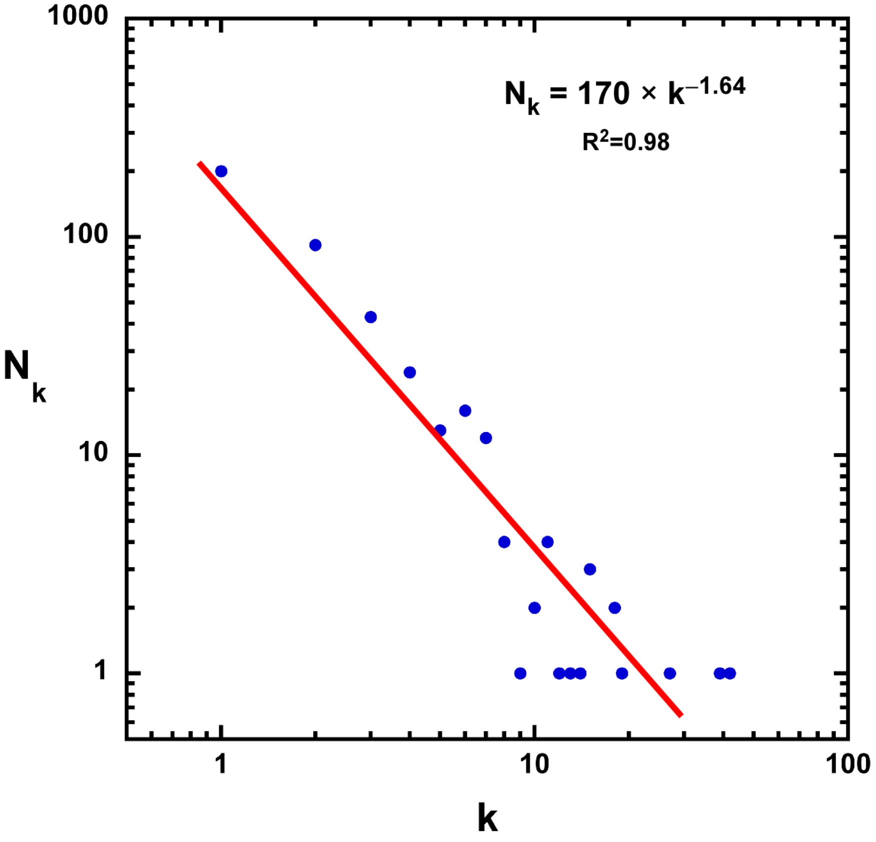

Supplement: Additional file 2 — Gene product interaction network of differentially expressed genes in VSELs. This file contains two figures depicting a gene product interaction network of differentially expressed genes in VSELs after in vivo exposure to hypoxia. Panel (A) highlights up and downregulated genes (red and green respectively). This network has 424 genes (nodes) and 604 connections (edges). Note that each connection represents a known direct interaction between two gene products. Panel (B) demonstrates that the topology of this network is scale-free because it follows a power law distribution. Nk, degree distribution; k, nodal connectivity. [file 1465-9921-12-63-S2.PDF]
